# Supplementary material for: Impact of COVID-19 pandemic on health service utilisation and household economy of pregnant and postpartum women: a cross-sectional study from rural Sri Lanka
Source: BMJ Open. 2023 May 29;13(5):e070214. doi: 10.1136/bmjopen-2022-070214 (PMC10230333; doi:10.1136/bmjopen-2022-070214)
Supplement: Supplementary data [file bmjopen-2022-070214supp001.pdf]

Rajarata University of Sri Lanka

Faculty of Medicine & Allied Sciences

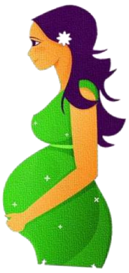

"Generating Evidence for Practice"

Rajarata Pregnancy Cohort (RaPCo)

Telephone Interview Guide

Maternal and Child Health Research Unit

Department of Community Medicine

Gunarathna SP, *et al. BMJ Open* 2023; 13:e070214. doi: 10.1136/bmjopen-2022-070214

Impact of COVID-19 Pandemic on Health Service Utilization and Household Economy of Pregnant and Postpartum Women: A Cross-Sectional Study from Rural Sri Lanka

Instructions for administering the questionnaire

- There are two main parts in this questionnaire. Part I (Economic Impact of COVID-19) includes five questions, and Part II (Health Service and Assistance during COVID-19 outbreak) contains nine questions.

- Most questions included here are close-ended; some were made for short answers and expected to be completed within 10-15 minutes.

- The interviewer must directly enter the answers to the questions below into the MS Excel data sheet.

- Please follow the following procedure to contact pregnant and postpartum women.

  First day - only two attempts should be made during the same day.

  Another day - Needs to be carried out for the women who could not contact during the first round, and only two attempts need to be made per day.

- After contacting, please introduce yourself and ask whether it is an appropriate time to talk over the phone. If not, politely apologize and ask for a possible time.

  “Good morning/Good afternoon!!! I am................................(name of the interviewer) and expect to conduct a telephone interview attached to the Faculty of Medicine and Allied Sciences, Rajarata University of Sri Lanka. We are conducting this survey to find out whether the COVID-19 pandemic affected the household economy and health service utilization of pregnant and postpartum women in rural Sri Lanka. Is this an appropriate time to talk?”

- Obtain verbal consent to conduct the interview and give those about two minutes to ask for any clarifications.

- If the respondent agrees to participate in the survey, start asking questions in the questionnaire. If the respondent refuses to participate in the survey at the beginning or middle of the interview, thank her and stop the discussion.

- Please read each question as written in the questionnaire and ask the questions in the order they appear.

Gunarathna SP, *et al. BMJ Open* 2023; 13:e070214. doi: 10.1136/bmjopen-2022-070214

- At the end of the questionnaire, thank the respondent for her cooperation and the information provided.

- At the end of the interview, it is essential to review the questionnaire to verify that all the questions were asked and each has an answer unless the respondent has refused to answer particular questions. If any question has been missed, you should ask it the relevant respondent again.

Questions

Part I – Economic Impact of COVID-19 Outbreak

1. What is your monthly income during the COVID-19 outbreak?
2. Did your family face any income reduction during the COVID-19 outbreak?
3. Could you please explain how much income was reduced monthly during the COVID-19 outbreak?
4. Did your family receive the financial aid of Rs.5,000.00 the Sri Lankan government provided monthly?
5. Can you state around how much did you or your family spend on monthly pregnancy-related nutritional expenditures?

Part II – Health Service and assistance during COVID-19 Outbreak

1. Did you miss clinic visits during the COVID-19 outbreak?
2. If yes, please indicate the number of missed clinics.
3. Did a Public Health Midwife (PHM) visit you or check up on phone calls during the COVID-19 outbreak?
4. If yes, how many times per month?
5. How was your satisfaction level with the service provided by PHM during the COVID-19 outbreak?
    - Strongly satisfied
    - Satisfied
    - Average

- Not satisfied
- Strongly unsatisfied

6. Was your mode of childbirth changed due to the COVID-19 outbreak?

7. If yes, what was your planned mode, and what was the changed mode?

8. Was your place of childbirth changed due to the COVID-19 outbreak?

9. How was your satisfaction level with the support from your husband/family and neighbours during the COVID-19 outbreak?

   - Strongly satisfied
   - Satisfied
   - Average
   - Not satisfied
   - Strongly unsatisfied
