# Supplementary material for: Impact of COVID-19 pandemic on health service utilisation and household economy of pregnant and postpartum women: a cross-sectional study from rural Sri Lanka
Source: BMJ Open. 2023 May 29;13(5):e070214. doi: 10.1136/bmjopen-2022-070214 (PMC10230333; doi:10.1136/bmjopen-2022-070214)
Supplement: Supplementary data [file bmjopen-2022-070214supp002.pdf]

Supplementary Table 1: Socio-demographic and economic characteristics of the study sample

Characteristic		Delivered during the COVID-19 pandemic (n=807, 55.3%)	Delivered before the COVID-19 pandemic (n=653, 44.7%)	Statistically significant difference/ associations*
Age of the pregnant women [Mean (SD)] in years		27.8 (5.1)	28.5 (5.9)	U=187,609.00 p=0.330
Ethnicity [n (%)]	Sinhalese	542 (85.1)	699 (86.9)	X^2 [df=1]=1.022 p=0.312
	Other minority ethnic groups[1]	95 (14.9)	105 (13.1)	
Religion [n (%)]	Buddhist	542 (85.1)	685 (85.2)	X^2 [df=1]=0.004 p=0.952
	Other religions[2]	95 (14.9)	119 (14.8)	
Educational status [n (%)]	Primary education	4 (0.7)	9 (1.2)	X^2 [df=3]=3.221 p=0.359
	Junior secondary education	21 (3.5)	39 (5.0)	
	Senior secondary education	484 (80.0)	612 (79.2)	
	Higher education	96 (15.9)	113 (14.6)	
Daily wage group [n (%)]	Yes	130 (21.0)	98 (18.7)	X^2 [df=1]=0.909 p=0.340
	No	489 (79.0)	425 (81.3)	
Income-generating members in the household	Only one person	683 (80.6)	379 (81.3)	X^2 [df=1]=0.093 p=0.760
	More than one person	164 (19.4)	87 (18.7)	
Income status in relation to the poverty line [n (%)][3]	Households below the national poverty line	105 (13.0)	68 (10.4)	X^2 [df=1]=0.632 p=0.260
	Households below the extreme poverty line	70 (8.7)	42 (6.4)	X^2 [df=1]=0.528 p=0.324
Per visit pregnancy expenditure during the first trimester (USD)	Mean (SD)	5.98 (7.77)	5.71 (7.50)	U=205,265.50 p=0.503
	Median (IQR)	4.05 (0.89-7.95)	3.59 (0.88-7.75)	
Per visit pregnancy expenditure during the second trimester	Mean (SD)	5.90 (6.99)	5.79 (6.90)	U=168,747.00 p=0.605
	Median (IQR)	3.50 (0.84-8.29)	3.48 (1.03-8.54)	

(USD)				

Note: [1]Tamil, Moor, Malays, and others, [2]Catholic/Christian, Hindu, Islam, and others, [3]Income status before COVID-19 pandemic, *The statistical significance difference/association of socio-demographic, economic, and health information was tested between women delivered during and before the COVID-19 pandemic, Mann-Whitney U test was used for continues variables, and Chi-square goodness of fit test was used for categorical variables
