# Supplementary material for: Impact of COVID-19 pandemic on health service utilisation and household economy of pregnant and postpartum women: a cross-sectional study from rural Sri Lanka
Source: BMJ Open. 2023 May 29;13(5):e070214. doi: 10.1136/bmjopen-2022-070214 (PMC10230333; doi:10.1136/bmjopen-2022-070214)
Supplement: Supplementary data [file bmjopen-2022-070214supp003.pdf]

Supplementary Table 2: COVID-19 impact on household income and status of receiving financial aids

Description			Delivered during COVID-19 pandemic [n=648, 55.4%]	Delivered before COVID-19 pandemic [n=524, 44.7%]
Impact on household income	Families affected by income reduction [n (%)]	Yes	342 (52.8)	250 (47.7)
		No	306 (47.2)	274 (52.3)
	Reduced amount (USD)	n (%)	342 (52.8)	250 (47.7)
		Mean (SD)	124.50 (85.19)	114.65 (76.93)
		Median (IQR)	106.20 (53.10-185.84)	106.20 (53.10-159.29)
	Percentage of reduced income over household income (%)		74.5	74.8
Monthly household income (USD) changes	n (%)		342 (52.8)	250 (47.7)
	Income before COVID-19	Mean (SD)	277.96 (194.07)	224.41 (179.46)
		Median (IQR)	212.39 (159.29-318.59)	185.84 (143.37-238.94)
	Income during COVID-19	Mean (SD)	172.89 (104.91)	154.85 (78.94)
		Median (IQR)	159.29 (106.20-238.94)	159.29 (106.20-212.39)
	Statistically significant difference[1]		Z=-6.317 p<0.001	Z=-6.314 p<0.001
Households pushed into poverty [n (%)]	National poverty line		31 (4.8)	25 (4.8)
	Extreme poverty line		27 (4.2)	20 (3.8)
Financial aid provided by the government (USD 26.55)	Received financial assistance among families pushed into poverty [n (%)]		0	2 (4.8)
	Received financial assistance among affected families [n (%)]		12 (3.5)	49 (19.6)
	Percentage of received financial aid over affected income (%)		47.5	42.7
	Percentage of received financial aid		14.4	28.4

	over household income (%)		
	Percentage of household expenditure covered by the received financial aid (%)	31.8	24.2

Note: [1]Wilcoxon signed ranks test
