# Supplementary material for: Impact of COVID-19 pandemic on health service utilisation and household economy of pregnant and postpartum women: a cross-sectional study from rural Sri Lanka
Source: BMJ Open. 2023 May 29;13(5):e070214. doi: 10.1136/bmjopen-2022-070214 (PMC10230333; doi:10.1136/bmjopen-2022-070214)
Supplement: Supplementary data [file bmjopen-2022-070214supp004.pdf]

Supplementary Table 3: Third trimester per visit pregnancy expenditure between women delivered during and before the COVID-19 pandemic

Cost [USD]	Summary Statistics	Delivered during COVID-19 Pandemic[1] [n=648, 55.4%]	Delivered before COVID-19 Pandemic[2] [n=524, 44.7%]	Statistically significant difference[3]
Cost of medicine	Mean (SD)	2.04 (3.08)	2.72 (3.76)	U=3,868.5
	Median (IQR)	1.06 (0.35-2.36)	1.42 (0.62-3.54)	p=0.050*
Cost for consultation	Mean (SD)	7.33 (3.84)	6.63 (3.08)	U=3,913.50
	Median (IQR)	6.90 (5.31-7.96)	6.70 (5.31-7.67)	p=1.164
Cost for laboratory investigation	Mean (SD)	2.17 (1.92)	2.82 (1.34)	U=48.0
	Median (IQR)	1.27 (0.93-2.65)	2.65 (2.02-3.85)	p=0.196
Cost for travelling	Mean (SD)	1.16 (2.02)	0.98 (1.32)	U=10,761.00
	Median (IQR)	0.53 (0.27-1.06)	0.53 (0.29-0.88)	p=0.941
Cost for food and refreshments	Mean (SD)	1.06 (1.29)	1.09 (1.39)	U=6,579.00
	Median (IQR)	0.66 (0.35-1.33)	0.66 (0.40-1.33)	p=0.668
Cost for accompanying person	Mean (SD)	0.85 (0.82)	1.39 (1.76)	U=925.5
	Median (IQR)	0.53 (0.40-0.93)	0.66 (0.53-1.33)	p=0.069*
Other costs	Mean (SD)	1.48 (1.86)	0.60 (0.50)	U=201.5
	Median (IQR)	0.53 (0.18-1.95)	0.60 (0.18-0.88)	p=0.181
Direct medical out-of-pocket expenditure	Mean (SD)	6.90 (6.34)	8.17 (6.72)	U=7,399.5
	Median (IQR)	6.64 (2.04-8.67)	7.06 (3.50-10.86)	p=0.095*
Direct non-medical out-of-pocket expenditure	Mean (SD)	1.94 (2.39)	2.29 (3.85)	U=14,406.0
	Median (IQR)	0.91 (0.53-2.27)	1.25 (0.44-2.43)	p=0.903
Per visit out-of-pocket expenditure in the 3rd trimester	Mean (SD)	7.26 (8.97)	7.01 (8.54)	U=19,668.00
	Median (IQR)	4.73 (1.02-9.64)	4.82 (0.73-10.19)	p=0.771

Note: [1]The cost indicated here was incurred during the COVID-19 pandemic, [2]The cost indicated here was incurred before the COVID-19 pandemic, [3]Mann-Whitney U test, *Statistically significant 0.1 level of significance
