# Supplementary material for: Impact of COVID-19 pandemic on health service utilisation and household economy of pregnant and postpartum women: a cross-sectional study from rural Sri Lanka
Source: BMJ Open. 2023 May 29;13(5):e070214. doi: 10.1136/bmjopen-2022-070214 (PMC10230333; doi:10.1136/bmjopen-2022-070214)
Supplement: Supplementary data [file bmjopen-2022-070214supp005.pdf]

Supplementary Table 4: Impact of COVID-19 on health service utilization of pregnant and postpartum women and support received by PHM and family

Description			Delivered during COVID-19 pandemic [n=732, 55.7%]	Delivered before COVID-19 pandemic [n=583, 44.3%]
Clinic visits[1]	Whether missed clinics [n (%)]	Yes	104 (14.2)	40 (6.9)
		No	628 (85.8)	543 (93.1)
	Number of missed clinics [median (IQR)]		1 (1-2)	1 (1-2)
Field health services from PHM	Whether PHM visited or did phone check-ups to see the pregnant women/infant [n (%)]	Yes	670 (91.5)	500 (85.8)
		No	62 (8.5)	83(14.2)
	Number of visits per month [median (IQR)]		2 (1-3)	2 (1-3)
	Service provided by the PHM [n (%)]	Strongly satisfied	4 (0.5)	3 (0.5)
		Satisfied	667 (91.2)	429 (73.6)
		Average	50 (6.8)	138 (23.7)
		Not satisfied	8 (1.1)	10 (1.7)
		Strongly not satisfied	3 (0.4)	3 (0.5)
Childbirth	Whether changed the mode of childbirth [n (%)]	Yes	4 (0.5)*	-
		No	728 (99.5)	-
	Whether changing the place of childbirth [n (%)]	Yes	14 (1.9)**	-
		No	718 (98.1)	-
Support from husband/family and neighbours received during the pandemic	Very satisfied		4 (0.5)	12 (2.1)
	Satisfied		650 (88.8)	442 (75.7)
	Average		56 (7.7)	105 (18.0)
	Dissatisfied		15 (2.0)	12 (2.1)

[n (%)]	Very dissatisfied	7 (1.0)	12 (2.1)

Note: [1]Indicated clinic visits are antenatal clinic visits for the women delivered during COVID-19 and postnatal/child health clinics for women delivered before COVID-19, *Number of pregnant women expected to change the MOD, **Number of pregnant women expected to change the place of childbirth
