# Supplementary material for: Impact of COVID-19 pandemic on health service utilisation and household economy of pregnant and postpartum women: a cross-sectional study from rural Sri Lanka
Source: BMJ Open. 2023 May 29;13(5):e070214. doi: 10.1136/bmjopen-2022-070214 (PMC10230333; doi:10.1136/bmjopen-2022-070214)
Supplement: Author's manuscript [file bmjopen-2022-070214.draft_revisions.pdf]

BMJ Open is committed to open peer review. As part of this commitment we make the peer review history of every article we publish publicly available.

When an article is published we post the peer reviewers' comments and the authors' responses online. We also post the versions of the paper that were used during peer review. These are the versions that the peer review comments apply to.

The versions of the paper that follow are the versions that were submitted during the peer review process. They are not the versions of record or the final published versions. They should not be cited or distributed as the published version of this manuscript.

BMJ Open is an open access journal and the full, final, typeset and author-corrected version of record of the manuscript is available on our site with no access controls, subscription charges or pay-per-view fees (http://bmjopen.bmj.com).

If you have any questions on BMJ Open's open peer review process please email info.bmjopen@bmj.com

BMJ Open

The COVID-19 Pandemic was Pushing the Rural Households with Pregnant and Postpartum Women into Poverty in Sri Lanka

Journal:	*BMJ Open*
Manuscript ID	bmjopen-2022-070214
Article Type:	Original research
Date Submitted by the Author:	18-Nov-2022
Complete List of Authors:	Gunarathne, Sajaan; Rajarata University of Sri Lanka Faculty of Medicine and Allied Sciences, Community Medicine<br>Wickramasinghe, Nuwan Darshana; Rajarata University of Sri Lanka Faculty of Medicine and Allied Sciences, Department of Community Medicine<br>Agampodi, Thilini; Rajarata University of Sri Lanka Faculty of Medicine and Allied Sciences, Department of Community Medicine<br>Prasanna, Indika Ruwan; Rajarata University of Sri Lanka, Department of Economics<br>Agampodi, Suneth; Rajarata University of Sri Lanka Faculty of Medicine and Allied Sciences, Department of Community Medicine
Keywords:	COVID-19, Health economics < HEALTH SERVICES ADMINISTRATION & MANAGEMENT, Public health < INFECTIOUS DISEASES

SCHOLARONE™
Manuscripts

I, the Submitting Author has the right to grant and does grant on behalf of all authors of the Work (as defined in the below author licence), an exclusive licence and/or a non-exclusive licence for contributions from authors who are: i) UK Crown employees; ii) where BMJ has agreed a CC-BY licence shall apply, and/or iii) in accordance with the terms applicable for US Federal Government officers or employees acting as part of their official duties; on a worldwide, perpetual, irrevocable, royalty-free basis to BMJ Publishing Group Ltd ("BMJ") its licensees and where the relevant Journal is co-owned by BMJ to the co-owners of the Journal, to publish the Work in this journal and any other BMJ products and to exploit all rights, as set out in our licence.

The Submitting Author accepts and understands that any supply made under these terms is made by BMJ to the Submitting Author unless you are acting as an employee on behalf of your employer or a postgraduate student of an affiliated institution which is paying any applicable article publishing charge ("APC") for Open Access articles. Where the Submitting Author wishes to make the Work available on an Open Access basis (and intends to pay the relevant APC), the terms of reuse of such Open Access shall be governed by a Creative Commons licence – details of these licences and which Creative Commons licence will apply to this Work are set out in our licence referred to above.

Other than as permitted in any relevant BMJ Author's Self Archiving Policies, I confirm this Work has not been accepted for publication elsewhere, is not being considered for publication elsewhere and does not duplicate material already published. I confirm all authors consent to publication of this Work and authorise the granting of this licence.

For peer review only

Title Page

Title

The COVID-19 Pandemic was Pushing the Rural Households with Pregnant and Postpartum Women into Poverty in Sri Lanka

Details of the corresponding author

Full Name: Sajaan Praveena Gunarathne

Postal address: Department of Community Medicine, Faculty of Medicine and Allied Sciences, Rajarata University of Sri Lanka, Saliyapura - Sri Lanka.

Email: sajaanpraveen7@gmail.com

Names and affiliations of the authors

1. Sajaan Praveena Gunarathne

Department of Community Medicine,

Faculty of Medicine and Allied Sciences,

Rajarata University of Sri Lanka,

Saliyapura - Sri Lanka.

2. Nuwan Darshana Wickramasinghe

Department of Community Medicine,

Faculty of Medicine and Allied Sciences,

Rajarata University of Sri Lanka,

Saliyapura - Sri Lanka.

3. Thilini Chanchala Agampodi

Department of Community Medicine,

For peer review only

Faculty of Medicine and Allied Sciences,

Rajarata University of Sri Lanka,

Saliyapura - Sri Lanka.

4. Indika Ruwan Prasanna

Department of Economics,

Faculty of Social Sciences and Humanities,

Rajarata University of Sri Lanka,

Mihintale – Sri Lanka

5. Suneth Buddhika Agampodi

Department of Community Medicine,

Faculty of Medicine and Allied Sciences,

Rajarata University of Sri Lanka,

Saliyapura - Sri Lanka.

For peer review only

Word Count (excluding title page, abstract, references, figures and tables): 4,038

The COVID-19 Pandemic was Pushing the Rural Households with Pregnant and Postpartum Women into Poverty in Sri Lanka

Abstract

Introduction: Pregnant women are considered a vulnerable group during the COVID-19 outbreak. We aim to describe how household economies and health service utilization of pregnant and postpartum women were affected during the pandemic.

Methods: This study was conducted in the Anuradhapura district, Sri Lanka, among pregnant and postpartum women recruited for the Rajarata Pregnancy Cohort (RaPCo) during the initial stage of the COVID-19 pandemic (from April to June 2020). Household economic and health service utilization details during the COVID-19 pandemic were gathered through telephone interviews. Socio-demographic and economic data were obtained from the cohort baseline and analyzed with descriptive and non-parametric analysis.

Results: Out of the 1,460 women in the sample, 55.3% (n=807) were pregnant, and 44.7% (n=653) were postpartum women. Of the total sample, 1,172 (80.3%) women participated in the economic component. The monthly household income was reduced [mean (SD) = USD 119.51 (81.61)] in 50.5% (n=592) families during the pandemic ($Z=-8.555, p<0.001$). Also, the nutritional expenditure of pregnant women was reduced ($Z=-2.023, p=0.043$) by 6.7%. A total of 120 (10.3%) families were pushed into poverty. Only 10.3% (n=61) of affected families had received financial aid from the government, which was only 31.0% and 40.4% of the monthly household income and expenditure, respectively. Pregnant women's families who were pushed into poverty were not received financial aid. The majority (n=1,096, 83.3%) were satisfied with the public health midwife's service.

Conclusion: During the early stages of the pandemic, healthcare utilization of pregnant women was minimally affected. Even before the country's current economic crisis, the household economies of pregnant women in rural Sri Lanka were severely affected, pushing families into poverty due to the pandemic. The impact of COVID-19 and the aftermath on pregnant women will have many consequences if the policies and strategies are not revised to cater for this issue.

Keywords: COVID-19 pandemic, Health services, Poverty, Pregnant women

Key Messages

- The COVID-19 pandemic is an unexpected global change that disrupted health service delivery and adversely affected the household economies, and pregnant women were recognized as a vulnerable group during the pandemic.
- The household economy of pregnant women was severely affected during the pandemic pushing most families into poverty.
- The nutrition expenditure of pregnant women was compromised during the pandemic.
- Antenatal and postnatal health service utilization was minimally affected in rural Sri Lanka in the initial phase of the pandemic, and a notable dedication of the field healthcare team to provide uninterrupted service was evident.
- A proper mechanism is needed to support and distribute government-led or other financial aid for affected families with pregnant women, especially considering the economic crisis as an aftermath of the pandemic.

Introduction

Sustainable Development Goals (SDG) aim to improve maternal and child health by challenging countries to reduce the global burden of maternal, newborn, and child mortality (1,2). However, disrupted healthcare service delivery, accessibility and poor healthcare utilization during the COVID-19 pandemic and the household economic crisis that followed in the aftermath impeded achieving the targets. It was an unexpected change in the global and national sociocultural and economic landscape affecting the daily well-being of households directly or indirectly (3,4). Many countries implemented stringent pandemic control measures, including national or local lockdowns (5), which led household economies into a crisis other than the effect on health service utilization (6). The World Health Organization (WHO) highlighted pregnant women as one of the vulnerable groups of the COVID-19 pandemic (7), particularly in resource-limited and lower-income countries (3,4).

Many countries recommended spacing visits while following up remotely by phone and hospital visits, only reserving emergency care (8). Besides, many countries have reported changes/reductions in maternal healthcare services during the COVID-19 pandemic. According to the available evidence, maternity care and birth practices changed in the United States of America (9). Nepal reported a reduction of institutional childbirth by more than half, with rises in neonatal mortality and poor quality of care (5). There has been a reduction in

For peer review only

health service utilization in North-East Ethiopia due to fear of pregnant women attending health institutions (3). In addition, poor access to healthcare care and reduced perceived social support for pregnant women were reported around the globe (10). Thus, the COVID-19 pandemic has adversely affected the utilization of maternal healthcare services.

Few studies have cited the adverse impact of the COVID-19 pandemic on the household economy (4,6), including adverse effects on household economies in Nigeria (11), changes in consumption patterns of Malaysian households (12), losing jobs and declining household income in some Asia-pacific countries—Cambodia, the Lao People's Democratic Republic, Malaysia, Indonesia, the Philippines, Myanmar, Thailand, and Vietnam, etc. (13). Additional economic burden during COVID-19 could be problematic for households of pregnant women since pregnancy expenditure was already being a burden in many low and middle-income countries (14). However, studies explicitly focusing on the COVID-19 impact on the household economy of pregnant women are lacking in the global evidence base.

In Sri Lanka, the studies on the COVID-19 impact on pregnant and postpartum women are scanty. Sri Lanka is a lower-middle-income country (15) where maternal health services are well developed and accessed by pregnant women via both the public (to a more significant extent) and the private health system (16,17). The government-free public health system delivers maternal care through a well-established network of primary health care officers, reporting 85.3% of pregnant women registered in recent years (18). Also, the country is among the leads of lower-middle-income countries regarding maternal and child health SDGs. Sri Lanka has reached the fourth phase of obstetric transition (19), and the healthcare system of Sri Lanka is an excellent example of universal public health system coverage, disease prevention through evidence-based strategies, and using culturally appropriate manners for health promotion, etc. (17). The solid network of public health personnel, especially the public health midwives (PHMs) that involve in domiciliary and clinic care in the field has been an asset to the system.

The first local index case emerged on 11th March 2020, and lockdowns/curfews were initiated after 17th March 2020 (20). During the COVID-19 pandemic's initial stage, a low mortality rate due to COVID-19 was recorded with the high public health system standards in Sri Lanka (20). The interim guidelines on maternal healthcare services were provided to sustain the services during the pandemic by the Family Health Bureau, Ministry of Health, the

national focal point of maternal healthcare in the Ministry of Health, Sri Lanka (21,22). It provided expert guidance on the procedures for organizing services, home visits, clinic services, communications, and protective measures (21). However, the COVID-19 pandemic caused a higher prevalence of anxiety and depression among pregnant women, requiring additional support from the family and field health officers (23). This could be worsened if the household economy was affected since the income status was associated with the pregnancy expenditure (24,25) and pregnancy nutritional status (26). Available evidence indicates that the COVID-19 pandemic created an economic meltdown at the household level and the Sri Lankan economy (27).

It is not well explored how much Sri Lankan maternal health services have been affected by the poor economic conditions of the household during the COVID-19 pandemic. The empirical studies explicitly focusing on the impact of the COVID-19 pandemic on maternal healthcare service utilization and the household economy of pregnant women in Sri Lanka are lacking. Assessing the effects of the COVID-19 epidemic on the household economy and health service utilization of pregnant and postpartum women would yield valuable evidence for decision-making during pandemic situations. Thus, this study aimed to evaluate the impact of the COVID-19 pandemic on the household economy and health service utilization of pregnant and postpartum women in rural Sri Lanka.

Methods

Study design and study setting

This cross-sectional study was conducted in the Anuradhapura district, the largest district in Sri Lanka (7,179 km^2), and was a part of the economic component of the largest pregnancy cohort conducted in Sri Lanka, the Rajarata Pregnancy Cohort (RaPCo) (28,29). The current population is about 900,000, and most (92.7%) belong to the district's rural population. The agriculture industry is the primary source of income, and the median monthly household income is USD 285.91 (30). Total health expenditure comprises 58% of government sources (31). Maternal, postpartum, and child health care is provided through 22 Medical Officer of Health (MOH) areas and 275 Public Health Midwife (PHM) areas in the government sector free of charge. Approximately 17,000 pregnant women register annually at the PHM, and the number of live births is about 15,000 (28). Further details on the study setting have already been published (28).

For peer review only

Study population and sample

We invited all pregnant women recruited for the RaPCo study from 1st July 2019 to 30th September 2019 to participate in this study. The eligibility criteria were as follows.

- Pregnant women registered at the PHM and visiting antenatal field clinics in the Anuradhapura district
- Pregnant women whose permanent residence in the Anuradhapura district had not planned to leave the district until childbirth
- Period of amenorrhoea/gestational age less than 12 weeks by the time of recruitment

The recruitment of pregnant women for the study was done with the help of PHMs in each MOH area by organizing a special clinic under the permission of the public health authorities in the district. Informed written consent was sought from the participants prior to the study. Ethical approval and administrative clearance were taken before the recruitment.

Data collection

With the permission of pregnant women during recruitment, we collected their contact numbers to contact them to share the investigation results and follow-up data collection. We conducted a telephone interview using a pre-tested interviewer guide with close-ended questions, and details on the routine utilization of antenatal/postnatal health services and household financial information during the pandemic were collected during the interviews (32). Five female medical graduates trained in telephone interview techniques, emphasizing politeness, telephone etiquette, basic counselling skills, and essential perinatal health information, carried out the interviews. If the first attempt failed, several attempts to contact pregnant women were made on the same day and subsequent days for the next two weeks. The second round of calls was done to contact those who were unavailable or busy the first time. Telephone interviews were conducted mainly during the pandemic and completed within three months (from April to June 2020). The duration of the interview was 4-10 minutes.

We explained the study objective and confirmed whether the study participation was voluntary. We obtained verbal consent during the interview apart from the written consent sorted at the recruitment in the RaPCo study. During the telephone interviews, we provided psychological support to pregnant and postpartum women where needed.

For peer review only

Data analysis

Data entering was done during the interviews using Microsoft Office Excel software, and then the data management was done using the same software. We combined the baseline data collected from the RaPCo study and the economic study with this database, and the analysis was carried out using SPSS V27.

Before the analysis, all the monetary values were converted to the average exchange rate (USD to Sri Lankan Rupees [LKR]) of the data collection period from April 2020 to June 2020 (i.e., USD 1 = LKR 188.33) (33). Using the average exchange rate for the above period was based on the fact that there were no outliers, and the standard deviation was only USD 2.99.

Further, we created a few variables, i.e., income reduction of ith household = income before COVID-19 pandemic of the ith household – income during COVID-19 pandemic of the ith household, percentage of affected income over household income of ith household = (reduced income of the ith household × 100%) / household income of the ith household. The same formula was used to create the percentage of received financial aid over affected income, the percentage of received financial aid over household income, and the percentage of received financial aid over household expenditure. Moreover, we used the national poverty line (USD 3.65), the poverty line of lower-middle-income countries (USD 3.20), and the extreme poverty line (USD 2.15) for creating new variables.

Sample characteristics (including socio-demographic, economic, and health-related information), health service utilization information, and household financial information during the pandemic were presented using univariate descriptive statistical measures. We presented the results in two groups; women who delivered during COVID-19 (after 17th March 2020) and women who delivered before COVID-19 (before 17th March 2020). We considered the date-17th March 2020 as the cut-off date to create this variable since the national lockdowns/curfews were started on 17th March 2020 (20).

We tested the normality of the data using the Shapiro-Wilk test before the bivariate analysis. The results revealed that the data was not normally distributed ($p<0.05$). Thus, non-parametric statistical tests were used for data analysis, including the Wilcoxon Signed Ranks test, Mann-Whitney U test, and Chi-square goodness of fit test.

For peer review only

Patient and public involvement

Patients and the public were not involved in the design, recruitment, conduct, reporting or dissemination plans of this research.

Results

Sample characteristics

Among the total sample (n=1,460), 44.7% (n=653) of women delivered before the COVID-19 pandemic and 55.3% (n=807) during the COVID-19 pandemic. The mean (SD) age of the pregnant and postpartum women was 28.2 (5.5) years. The majority were Sinhalese (n=1,250, 85.6%) and Buddhists (n=1,237, 84.7). Most women (n=1,162, 79.6%) were educated up to senior secondary education. In most families (n=1,180, 80.8%), only one person was involved in regular income-generating activities, and 27.6 (n=409) were below the poverty line (Table 1).

Table 1: Socio-demographic and economic characteristics of the study sample

Characteristic		Delivered during the COVID-19 pandemic (n=807, 55.3%)	Delivered before the COVID-19 pandemic (n=653, 44.7%)	Total (n=1,460, 100%)
Age of the pregnant women [Mean (SD)] in years		27.8 (5.1)	28.5 (5.9)	28.2 (5.5)
Ethnicity [n (%)]	Sinhalese	687 (85.1)	568 (87.0)	1,250 (85.6)
	Other minority ethnic groups[1]	120 (14.9)	85 (13.0)	210 (14.4)
Religion [n (%)]	Buddhist	627 (77.7)	558 (85.5)	1,237(84.7)
	Other religions[2]	180 (22.3)	95 (14.3)	223 (15.3)
Educational status [n (%)]	Primary education	5 (0.6)	8 (1.2)	15 (1.0)
	Junior secondary education	28 (3.5)	33 (5.1)	63 (4.3)
	Senior secondary education	646 (80.0)	517 (79.2)	1,162 (79.6)
	Higher education	128 (15.9)	95 (14.5)	220 (15.1)
Daily wage group [n (%)]	Yes	169 (20.9)	122 (18.7)	296 (20.3)
	No	638 (79.1)	531 (81.3)	1,164 (79.7)

For peer review only

Income-generating members in the household	Only one person	715 (88.6)	531 (81.3)	1,180 (80.8)
	More than one person	92 (11.4)	122 (18.7)	280 (19.2)
Income status in relation to the poverty line [n (%)][3]	Households below the national poverty line	102 (12.6)	62 (9.5)	164 (11.2)
	Households below the lower-middle income countries' poverty line	98 (12.1)	56 (8.6)	154 (10.5)
	Households below the extreme poverty line	61 (7.6)	30 (4.6)	91 (6.2)
Per visit pregnancy expenditure during the first trimester (USD)	Mean (SD)	5.98 (7.77)	5.71 (7.50)	5.82 (7.60)
	Median (IQR)	4.05 (0.89-7.95)	3.59 (0.88-7.75)	3.72 (0.88-7.88)
Per visit pregnancy expenditure during the second trimester (USD)	Mean (SD)	5.90 (6.99)	5.79 (6.90)	5.82 (7.00)
	Median (IQR)	3.50 (0.84-8.29)	3.48 (1.03-8.54)	3.45 (0.88-8.50)

Note: [1]Tamil, Moor, Malays, and others, [2]Catholic/Christian, Hindu, Islam, and others, [3]Income status before COVID-19 pandemic

Impact of COVID-19 pandemic on household economy

The pregnant and postpartum women's monthly household income showed a statistically significant difference (p<0.001) during the pandemic and the pre-COVID period. Monthly household income had been reduced during the COVID-19 period compared to the pre-COVID period. The household income was affected in 50.5% (n=592) of families, and the percentage of reduced income in terms of household income was 74.6%. Even though monthly household income was reduced, there was no statistically significant difference (Z=-0.827, p=0.408) in household expenditure during and before the COVID-19 pandemic for the families of women delivered during COVID-19 (n=807, 55.3%).

The majority (n=1,111, 94.8%) had not received the government's financial aid, and the obtained amount covered only 46.4% of the affected income (Table 2).

Table 2: COVID-19 impact on household income and status of receiving financial aids

Description	Delivered during COVID-19	Delivered before COVID-19	Total [n=1,172, 100%]

For peer review only

			**pandemic [n=648, 55.4%]**	**pandemic [n=524, 44.7%]**	
Impact on household income	Families affected by income reduction [n (%)]	Yes	342 (52.8)	250 (47.7)	592 (50.5)
		No	306 (47.2)	274 (52.3)	580 (49.5)
	Reduced amount (USD)	n (%)	342 (52.8)	250 (47.7)	592 (50.5)
		Mean (SD)	124.50 (85.19)	114.65 (76.93)	119.51 (81.61)
		Median (IQR)	106.20 (53.10-185.84)	106.20 (53.10-159.29)	106.20 (53.10-159.29)
	Percentage of reduced income over household income (%)		74.5	74.8	74.6
Monthly household income (USD) changes	n (%)		342 (52.8)	250 (47.7)	592 (50.5)
	Income before COVID-19	Mean (SD)	277.96 (194.07)	224.41 (179.46)	251.08 (188.45)
		Median (IQR)	212.39 (159.29-318.59)	185.84 (143.37-238.94)	212.39 (159.29-265.49)
	Income during COVID-19	Mean (SD)	172.89 (104.91)	154.85 (78.94)	164.30 (95.11)
		Median (IQR)	159.29 (106.20-238.94)	159.29 (106.20-212.39)	159.29 (106.20-212.39)
	Statistically significant difference[1]		Z=-6.317 p<0.001	Z=-6.314 p<0.001	Z=-8.555 p<0.001
Households pushed into poverty [n (%)]	National poverty line		28 (4.3)	16 (3.1)	44 (3.8)
	Lower-middle income country's poverty line		29 (4.5)	18 (3.4)	47 (4.0)
	Extreme poverty line		21 (3.2)	8 (1.5)	29 (2.5)
Financial aid provided by the government (USD 26.55)	Received financial assistance among families pushed into poverty [n (%)]		0	2 (4.8)	2 (1.7)
	Received financial assistance among affected families [n		12 (3.5)	49 (19.6)	61 (10.3)

For peer review only

	(%)]			
	Percentage of received financial aid over affected income (%)	47.5	42.7	46.4
	Percentage of received financial aid over household income (%)	14.4	28.4	17.9
	Percentage of household expenditure covered by the received financial aid (%)	31.8	24.2	29.9

Note: [1]Wilcoxon signed ranks test

Daily wage earners' (n=296, 20.3%) income was reduced by 80.0%. Only 14.5% (n=43) of families in the daily wage group have received government financial aid (USD 26.55), covering 57.6% of affected income, 19.1% of household income, and 33.5% of household expenditure.

Among the total sample, a considerable number of families (n=120, 10.3%) were pushed into poverty and extreme poverty due to the pandemic. The total affected amount [mean (SD) = USD 119.51 (81.61), median (IQR) = USD 106.20 (53.10-159.29)] was equivalent to 72.7% of their monthly household income. However, only 10.3% (n=61) of affected families had received financial aid from the government, which was only 54.3% of the affected income, 31.0% of the monthly household income, and 40.4% of the monthly household expenditure. Further financial aid was not received for families with pregnant women who were pushed into poverty; in total, only 1.7% (n=2) of families who were pushed into poverty received financial aid.

Impact on pregnancy expenditure and nutritional expenditure

We compared the third-trimester antenatal care cost between the women delivered during and before the COVID-19 pandemic (Table 3). The third-trimester pregnancy cost for the women delivered before the COVID-19 period was used as the comparison group to test the cost incurred during the COVID-19 pandemic since there is not a baseline difference-there were no statistically significant differences in all the socio-demographic and economic variables between the women delivered during and before the COVID-19 pandemic (p>0.05) [Supplementary Table 1].

For peer review only

There was no statistically significant difference in spending per visit during the COVID-19 period compared to pre-COVID-19. However, there was a statistical significance difference ($p<0.1$) between the cost of medicine, the cost for accompanying persons, and direct medical cost within the two groups.

Further, there was a statistically significant difference (Z=-2.023, p=0.043) in nutritional expenditure during the third trimester [mean (SD) = USD 56.69 (33.79), median (IQR) = USD 53.10 (37.17-79.65)] compared with the early pregnancy nutritional expenditure [mean (SD) = USD 60.75 (33.40), median (IQR) = USD 54.43 (37.17-79.65)] of women delivered during COVID-19 pandemic (n=807, 55.3%). The reduction of nutritional expenditure was 6.7% compared with early pregnancy.

Table 3: Third trimester per visit pregnancy expenditure between women delivered during and before the COVID-19 pandemic

Cost [USD]	Summary Statistics	Delivered during COVID-19 Pandemic[1] [n=648, 55.4%]	Delivered before COVID-19 Pandemic[2] [n=524, 44.7%]	Statistically significant difference[3]
Cost of medicine	Mean (SD)	2.04 (3.08)	2.72 (3.76)	U=3,868.5
	Median (IQR)	1.06 (0.35-2.36)	1.42 (0.62-3.54)	p=0.050*
Cost for consultation	Mean (SD)	7.33 (3.84)	6.63 (3.08)	U=3,913.50
	Median (IQR)	6.90 (5.31-7.96)	6.70 (5.31-7.67)	p=1.164
Cost for laboratory investigation	Mean (SD)	2.17 (1.92)	2.82 (1.34)	U=48.0
	Median (IQR)	1.27 (0.93-2.65)	2.65 (2.02-3.85)	p=0.196
Cost for travelling	Mean (SD)	1.16 (2.02)	0.98 (1.32)	U=10,761.00
	Median (IQR)	0.53 (0.27-1.06)	0.53 (0.29-0.88)	p=0.941
Cost for food and refreshments	Mean (SD)	1.06 (1.29)	1.09 (1.39)	U=6,579.00
	Median (IQR)	0.66 (0.35-1.33)	0.66 (0.40-1.33)	p=0.668
Cost for accompanying person	Mean (SD)	0.85 (0.82)	1.39 (1.76)	U=925.5
	Median (IQR)	0.53 (0.40-0.93)	0.66 (0.53-1.33)	p=0.069*
Other costs	Mean (SD)	1.48 (1.86)	0.60 (0.50)	U=201.5
	Median (IQR)	0.53 (0.18-1.95)	0.60 (0.18-0.88)	p=0.181
Direct medical out-of-pocket expenditure	Mean (SD)	6.90 (6.34)	8.17 (6.72)	U=7,399.5
	Median (IQR)	6.64 (2.04-8.67)	7.06 (3.50-10.86)	p=0.095*
Direct non-medical out-	Mean (SD)	1.94 (2.39)	2.29 (3.85)	U=14,406.0

of-pocket expenditure	Median (IQR)	0.91 (0.53-2.27)	1.25 (0.44-2.43)	p=0.903
Per visit out-of-pocket expenditure in the 3rd trimester	Mean (SD)	7.26 (8.97)	7.01 (8.54)	U=19,668.00 p=0.771
	Median (IQR)	4.73 (1.02-9.64)	4.82 (0.73-10.19)	

Note: [1]The cost indicated here was incurred during the COVID-19 pandemic, [2]The cost indicated here was incurred before the COVID-19 pandemic, [3]Mann-Whitney U test, *Statistically significant 0.1 level of significance

Impact of COVID-19 on routine health service utilization of pregnant and postpartum women

Of the total sample, 14.2% (n=104) of pregnant women and 6.9% (n=40) of postpartum women missed clinics due to the COVID-19 pandemic. The median (IQR) number of missed clinics was 1 (1-2). The majority (n=1,096, 83.3%) of the pregnant and postpartum women were satisfied with the service provided by the PHM. Most women (n=1,170, 89.0%) visited/received phone check-ups by PHM, and the median (IQR) visits/phone checkups were 2 (1-3). Furthermore, most pregnant (n=1,092, 83.1%) and postpartum women were satisfied with the support/assistance received by the spouse/family members and neighbours during the COVID-19 pandemic. Only 0.5% (n=4) of pregnant women had to change the mode of childbirth, and 1.9% (n=14) women had to change the place of childbirth due to the travel restrictions of the curfew period (Table 4).

There is no association between the health service utilization of pregnant women and the income changes during the COVID-19 pandemic [X^2(df=1)=0.361,p=0.548].

Table 4: Impact of COVID-19 on health service utilization of pregnant and postpartum women and support received by PHM and family

Description			Delivered during COVID-19 pandemic [n=732, 55.7%]	Delivered before COVID-19 pandemic [n=583, 44.3%]	Total [n=1,315, 100%]
Clinic visits[1]	Whether missed clinics [n (%)]	Yes	104 (14.2)	40 (6.9)	144 (11.0)
		No	628 (85.8)	543 (93.1)	1,171 (89.0)
	Number of missed clinics [median (IQR)]		1 (1-2)	1 (1-2)	1 (1-2)

For peer review only

Field health services from PHM	Whether PHM visited or did phone check-ups to see the pregnant women/infant [n (%)]	Yes	670 (91.5)	500 (85.8)	1,170 (89.0)
		No	62 (8.5)	83(14.2)	145 (11.0)
	Number of visits per month [median (IQR)]		2 (1-3)	2 (1-3)	2 (1-3)
	Service provided by the PHM [n (%)]	Strongly satisfied	4 (0.5)	3 (0.5)	7 (0.5)
		Satisfied	667 (91.2)	429 (73.6)	1,096 (83.3)
		Average	50 (6.8)	138 (23.7)	188 (14.3)
		Not satisfied	8 (1.1)	10 (1.7)	18 (1.4)
		Strongly not satisfied	3 (0.4)	3 (0.5)	6 (0.5)
Childbirth	Whether changed the mode of childbirth [n (%)]	Yes	4 (0.5)*	-	-
		No	728 (99.5)	-	-
	Whether changing the place of childbirth [n (%)]	Yes	14 (1.9)**	-	-
		No	718 (98.1)	-	-
Support from husband/family and neighbours received during the pandemic [n (%)]	Very satisfied		4 (0.5)	12 (2.1)	16 (1.2)
	Satisfied		650 (88.8)	442 (75.7)	1,092 (83.1)
	Average		56 (7.7)	105 (18.0)	161 (12.2)
	Dissatisfied		15 (2.0)	12 (2.1)	27 (2.1)
	Very dissatisfied		7 (1.0)	12 (2.1)	19 (1.4)

Note: [1]Indicated clinic visits are antenatal clinic visits for the women delivered during COVID-19 and postnatal/child health clinics for women delivered before COVID-19, *Number of pregnant women expected to change the MOD, **Number of pregnant women expected to change the place of childbirth

Discussion

For peer review only

This study assessed the impact of the COVID-19 pandemic on health service utilization and the household economy of pregnant and postpartum women in rural Sri Lanka. We found that the household income has been reduced by 74.6% in most families, and 120 families (10.3%) were pushed below the poverty line. We identified that the most vulnerable group was daily income earners, and the financial aid received was two fifth-a small share compared with the income loss. Further, no changes in per-visit pregnancy expenditure between pregnant women delivered during and before the COVID-19 period. However, minor differences were highlighted in the cost of medicine, the cost for accompanying persons, and direct medical expenditures. Moreover, we found a reduction in the nutritional expenditure of pregnant women during the COVID-19 period. Health services were minimally affected, and health service utilization was not associated with income reduction. Further, the services provided by PHM were highly appreciated by pregnant and postpartum women

Impact on household economy and pregnancy expenditure

Even though it is the early stage of the COVID-19 pandemic, most households' income was affected by the local and national pandemics. Almost half of the families experienced an income reduction; the reduced income was equivalent to three-quarters of the monthly household income. Moreover, the severity of the pandemic has shown since a considerable amount of families were pushed into poverty (n=91, 7.8%) and extreme poverty (n=29, 2.5%). The most vulnerable group was daily income earners, who were one-fifth of the total sample. This is because of unavailability and the inability to seek work during the pandemic due to pandemics and curfews (34). Daily income earners must spend 70.0% of per day's income per visit to antenatal care in rural Sri Lanka (25). This could impact pregnant women's mental health as experienced higher anxiety in Pakistan's pregnant and postpartum women due to the lack of healthcare utilization due to more significant financial problems during the COVID-19 pandemic (35). By looking at the global level evidence, household income reduction and poverty due to the pandemic were experienced in China (36), countries in the European Union (37), and India (38).

Food insecurity and changes in food consumption patterns due to income reduction during the COVID-19 pandemic were recorded in a multi-country survey (39), Kenya and Uganda (40), Indonesia (41), Tajikistan (42), and Thailand (43). Although nutritional status is considered important in pregnancy, there was a reduction in nutritional expenditure compared with early pregnancy due to income reduction. This could be a sensitive matter since 16.5%

For peer review only

of pregnant women were recorded as underweight, and 14.5% of anaemic women were in the sample and also as reported in other studies conducted in the same study setting (44–46). It is also evident that household food diversity and nutritious food intake are compromised at the early stage of food insecurity in the same population of pregnant women even before the pandemic (47). Hence, necessary policies and mechanisms must be implemented to safeguard the food security of pregnant women at-risk during similar situations.

The burden of out-of-pocket expenditure has been highlighted despite accessing free services from government health facilities in India during the COVID-19 pandemic (48). However, with the organized and freely provided government maternal health care, the pregnancy expenditure was expected to be at a lower level compared to the pre-COVID-19 period since the pregnancy out-of-pocket expenditure showed a decline of 16.2% around the globe (49). However, the pregnancy expenditure of the pregnant women delivered during the COVID-19 pandemic did not differ from the expenditure incurred for pregnant women delivered before the COVID-19 pandemic. Only a slight decline showed in the cost of medicine and cost for accompanying person. Pregnancy expenditure is identified as an economic burden in the same study setting since it exceeds more than 10% of household income (24,25). This is problematic for the households whose income was affected, pushed into poverty, and the pregnancy and household expenditure remained unchanged during the pandemic.

Although the Sri Lankan government provided low-income people with financial aid (USD 26.55), pregnant women's families who were pushed into poverty were not received financial assistance and only 1.7% of families who were pushed into poverty received financial aid. However, only 10.3% of the affected families have received, while 27.6% were already below the poverty line, adding more than 10.3% into poverty due to the pandemic. Also, financial aid received a small share compared to income loss and household expenditures during the COVID-19 pandemic.

Health service utilization during the pandemic

During the COVID-19 pandemic, routine clinics were limited in Sri Lanka due to local/national pandemics and curfews to minimize the spread of the disease (20). However, compared to the other countries, the support received from spouses/family and neighbours and the antenatal and postnatal healthcare were at a satisfactory level since reporting poor access to antenatal care and reduced perceived family/social support in the United States of America, Ireland and United Kingdom (10). Further evidence of missing antenatal and

For peer review only

postnatal care was reported in Saudi Arabia (50), Sub-Saharan Africa (51), India (52), Pakistan (35), and almost all middle-income countries (53). However, the present study reports that most pregnant and postpartum women have received at least two phone check-ups per month or visited by the PHM during the pandemic. This could be due to the maternal health services provided even under the imposed restrictions during the pandemic. Sri Lanka has a unique public health system delivering maternal care through a well-established network of primary health care officers. This is unique because it provides free domiciliary care and clinic-based service through grass-root level health officers, mainly via PHMs (28). During the COVID-19 pandemic, health authorities in Sri Lanka provided interim guidelines highlighting that the PHM needs to do domiciliary visits to the infection control procedures and contact over the phone (21,22). Also, clinical services were provided at the MOH office via making appointments through PHM, especially for pregnant women with 36 weeks of POA or above. Emergency services were allocated for pregnant and postpartum women who needed immediate care through the interim guidelines (22). These could be the possible reasons for receiving the required health care for most pregnant women and stating a higher satisfaction level for the service provided by the PHM during the pandemic. This is appreciated since this period was particular, and the typical situation dramatically changed during this period, where the PHMs were utilized for vaccine delivery during the pandemic.

Moreover, identified 'no association' between the adverse impact of the household economy and health service utilization could result from this freely provided services by the Sri Lankan health system. Also, the available evidence of the region reported that families of pregnant women compromise other expenditures regarding pregnancy-related matters, and even low-income families save money for pregnancy needs (54). However, due to the travel restrictions, 14 pregnant women had to change the planned palace of childbirth to the nearest health institution.

Strengths and limitations of the study

We conducted telephone interviews of pregnant and postpartum women with a higher sample size compared to a lower sample in other related studies conducted on the same subject. We combined telephone interview data, baseline and economic data of the RaPCo study to see the changes in the household economy.

Even though telephone interviews and remote data collection and health promotion methods sound promising during the pandemic, conducting a telephone interview could lead to a

For peer review only

selection bias where a lack of accessibility of the communication devices and poor connection facilities. This also could lead to reducing the data quality and validity.

Conclusions

Despite having an adverse impact on the household economy and healthcare delivery, the healthcare utilization of pregnant women was minimally affected during the early stage of the COVID-19 pandemic in rural Sri Lanka, which was mainly attributed to the sustained delivery of maternal and child health services with a satisfactory service provided by the field public healthcare workers. Despite being in the early stage of the pandemic, household economies were severely affected and pushed families into poverty and extreme poverty. The adverse economic impact caused a reduction in the nutritional expenditure for pregnant women. The financial aid was not received by all having economic hardship, and the obtained amount was insufficient compared to the loss and household expenditure. While these observations were made on the impact in the initial phase, the aftermath of COVID-19 and the subsequent economic crisis in Sri Lanka may have a massive effect on pregnant women.

Unless policies and strategies are implemented to protect and maintain the maternal healthcare provision under these circumstances, the globally appreciated Sri Lankan maternal health system will face an imminent threat. Government and international aid during the post-pandemic economic crisis must be well-targeted and strategic to provide equitable care for pregnant women. This paper presents only the immediate impact of the COVID-19 pandemic during its early stages. Therefore, the current status, which rapidly changes, requires further studies to see the long-term impact of the pandemic.

Contributorship statement: SPG, NDW and SBA were responsible for the study concept and design. SPG and TCA did the data and project management. SPG did the data cleaning and analysis. SPG drafted and interpreted the data and drafted the manuscript. NDW, IRP, TCA and SBA reviewed the manuscript. SPG is responsible for the overall content as the guarantor. All the authors approved the final manuscript as submitted and agree to be accountable for all aspects of the work.

Competing Interests: The authors declare that they have no competing interests.

Funding: This research was supported by the Accelerating Higher Education Expansion and Development (AHEAD) Operation of the Ministry of Higher Education, Sri Lanka, funded by the World Bank. The funding agency has no role in the study's design, collection,

For peer review only

analysis, interpretation of data, and manuscript writing. The grant number is DOR STEM HEMS [6026-LK/ 8743-LK].

Data sharing statement: Data of the present study are from the *anonymized* study and are not publically available. The datasets used and/or analyzed during the current study are available from the corresponding author on reasonable request.

Ethical approval: This study was conducted under the large cohort study in Anuradhapura District, Sri Lanka, *anonymized* Cohort. Ethical clearance to conduct the *anonymized* study was obtained from the Ethics Review Committee of the Faculty of Medicine and Allied Sciences, Rajarata University of Sri Lanka (Reference no: ERC/2019/07). In accordance with the Declaration of Helsinki, all participants were informed that this study was voluntary and that data would be handled confidentially. All participants gave their informed verbal consent.

Acknowledgements: We acknowledge Dr Imasha Jayasinghe, Department of Community Medicine, Faculty of Medicine and Allied Sciences, Rajarata University of Sri Lanka, for the extensive support during the data collection.

References

1. Kisiangani I, Elmi M, Bakibinga P, Mohamed SF, Kisia L, Kibe PM, et al. Persistent barriers to the use of maternal, newborn and child health services in Garissa sub-county, Kenya: A qualitative study. BMC Pregnancy Childbirth. 2020;20(1):1–12.

2. Akaba G, Dirisu O, Okunade K, Adams E, Ohioghame J, Obikeze O, et al. Impact of COVID-19 on utilization of maternal, newborn and child health services in Nigeria: protocol for a country-level mixed-methods study. F1000Research. 2020;9:1106.

3. Assefa KT, Gashu AW, Mulualem TD. The impact of COVID-19 infection on maternal and reproductive health care services in governmental health institutions of Dessie town, North-East Ethiopia, 2020 G.C. medRxiv. 2020;2020.09.20.20198259.

4. Menendez, Clara; Gonzalez, Raquel ; Donnay F, Leke RGF. Avoiding indirect effects of COVID-19 on maternal and child health. Lancet Glob Heal. 2020;(January):19–21.

5. KC A, Gurung R, Kinney M V., Sunny AK, Moinuddin M, Basnet O, et al. Effect of the COVID-19 pandemic response on intrapartum care, stillbirth, and neonatal

For peer review only

mortality outcomes in Nepal: a prospective observational study. Lancet Glob Heal. 2020;8(10):e1273–81.

6. Martin A, Markhvida M, Hallegatte S, Walsh B. Socio-Economic Impacts of COVID-19 on Household Consumption and Poverty. Econ Disasters Clim Chang. 2020;4(3):453–79.

7. World Health Organization (WHO). Coronavirus disease (COVID-19): Pregnancy and childbirth. 2020.

8. Abdelbadee AY, Abbas AM. Impact of COVID-19 on reproductive health and maternity services in low resource countries. Eur J Contracept Reprod Heal Care. 2020;25(5):402–4.

9. Davis-Floyd R, Gutschow K, Schwartz DA. Pregnancy, Birth and the COVID-19 Pandemic in the United States. Med Anthropol Cross Cult Stud Heal Illn. 2020;39(5):413–27.

10. Meaney S, Leitao S, Olander EK, Pope J, Matvienko-Sikar K. The impact of COVID-19 on pregnant womens' experiences and perceptions of antenatal maternity care, social support, and stress-reduction strategies. Women and Birth [Internet]. 2022;35(3):307–16. Available from: https://doi.org/10.1016/j.wombi.2021.04.013

11. Fatoye F, Gebrye T, Arije O, Fatoye CT, Onigbinde O, Mbada CE. Economic Impact of COVID-19 lockdown on households. Pan Afr Med J [Internet]. 2021;40(225):1–9. Available from: https://www.panafrican-med-journal.com//content/article/40/225/full

12. Ismail MK, Kumaran VV, Munawwarah SN, Muhamad MZ, Sarifuddin S. COVID-19 Outbreak: An Analysis of Malaysian Household Income Class during Movement Control Order (MCO). Estud Econ Apl. 2021;39(4).

13. Morgan PJ, Trinh LQ. Impacts of COVID-19 on Households in ASEAN Countries and Their Implications for Human Capital Development [Internet]. Asian Development Bank Institute - ADBI Working Paper Series. 2021. Available from: https://www.adb.org/publications/impacts-covid-19-households-asean-countries

14. Gunarathne SP, Wickramasinghe ND, Agampodi TC, Prasanna IR, Agampodi SB. The magnitude of out-of-pocket expenditure for antenatal care in low and middle-income

For peer review only

countries: A systematic review and meta-analysis. Int J Health Plann Manage. 2022;(August):1–25.

15. World Bank Group. World Bank Country and Lending Groups. Country Classification. 2019. p. 7677.

16. Family Health Bureau: Ministry of Health. Maternal Care Package: A Guide to Field Health Care Workers. 1st ed. Colombo: Family Health Bureau, Ministry of Health, Sri Lanka; 2011.

17. Agampodi T, Amarasinghe G, Wickramasinghe N, Warnasekara J, Perez-Escamilla R, Agampodi S. Maternal and child health and nutrition (MCHN) policies and programs in Sri Lanka: a paradigm for public health in Asia. Sri Lanka J Med. 2021;30(2):70–87.

18. Family Health Bureau: Ministry of Health - Sri Lanka. Percentage of pregnant mothers registered in 2021 [Internet]. 2022 [cited 2022 Oct 22]. Available from: https://fhb.health.gov.lk/index.php/en/

19. Souza J, Tunçalp Ö, Vogel J, Bohren M, Widmer M, Oladapo O, et al. Obstetric transition: the pathway towards ending preventable maternal deaths. BJOG An Int J Obstet Gynaecol. 2014;121:1–4.

20. Arambepola C, Wickramasinghe ND, Jayakody S, Hewage SA, Wijewickrema A, Gunawardena N, et al. Sri Lanka's early success in the containment of COVID-19 through its rapid response: Clinical & epidemiological evidence from the initial case series. PLoS One [Internet]. 2021;16(7 July):1–13. Available from: http://dx.doi.org/10.1371/journal.pone.0255394

21. Family Health Bureau: Ministry of Health and Indigenous Medical Sevice SL. Interim guidlines on maternal and child health services during CURFEW PERIOD during outbreak of COVID-19 infection (Date:2020 March 27). Colombo, Sri Lanka; 2020.

22. Family Health Bureau: Ministry of Health and Indigenous Medical Sevice SL. Annual Report on Family Health Family Health Bureau. Colombo, Sri Lanka; 2018.

23. Patabendige M, Wanniarachchi D, Weerasinghe M, Ruwanpathirana P, Jayasundara D, Jayawardane A. The sustained adverse impact of COVID-19 pandemic on mental

For peer review only

health among pregnant women in Sri Lanka: a reassessment during the second wave. BMC Res Notes [Internet]. 2022;15(1):15–9. Available from: https://doi.org/10.1186/s13104-021-05893-1

24. Gunarathne SP, Wickramasinghe ND, Agampodi TC, Prasanna IR, Agampodi SB. How costly is the first prenatal clinic visit ? Analysis of out-of-pocket expenditure in rural Sri Lanka - a country with free maternal health care. BMC Health Serv Res. 2021;21(974):1–13.

25. Gunarathne, Sajaan Praveena Wickramasinghe ND, Agampodi TC, Prasanna IR, Agampodi SB. Out-of-pocket Expenditure for Antenatal Care Instills amidst Free Health Care Provision : Evidence from a Large Pregnancy Cohort in Rural Sri Lanka. Res Sq Pri-print. 2022;1–19.

26. Gunarathne S, Wickramasinghe N, Agampodi T, Prasanna R, Agampodi S. Economic Status, Nutrition and Pregnancy Cost; A Vicious Cycle in Pregnancy. Curr Dev Nutr [Internet]. 2020;4(2):996. Available from: https://academic.oup.com/cdn/article/4/Supplement_2/996/5844748?login=false

27. Rusniya H., Nufile AAM. The Impact of COVID-19 on Sri Lankan Economy. Int Res J Fac Arts Cult. 2021;13(4):11–9.

28. Agampodi TC, Wickramasinghe ND, Prasanna RIR, Irangani MKL, Banda JMS, Jayathilake PMB, et al. The Rajarata Pregnancy Cohort (RaPCo): study protocol. BMC Pregnancy Childbirth. 2020;20(1):374.

29. Gunarathne SP, Wickramasinghe ND, Agampodi TC, Prasanna IR, Agampodi SB. Economic burden of out-of-pocket expenditure, productivity cost during pregnancy and COVID-19 impact on household economy in a cohort of pregnant women in Anuradhapura District, Sri Lanka; A study protocol. F1000Research. 2022;10(May):1–20.

30. Department of Census and Statistics of Sri Lanka. Household Income and Expenditure Survey 2016 Final Report. 2018. 6 p.

31. Institute for Health Policy of Sri Lanka. Data and Statistics. 2020. p. 2020.

32. Jayasinghe I, Wickramasinghe Y, Kurera DM, Samarakoon N, Rupasinghe I,

For peer review only

Ponmalay P, et al. Feasibility of using telephone interviews and internet-based message services during the COVID-19 pandemic in rural Sri Lanka: experiences of the Rajarata Pregnancy Cohort. Rural Remote Health. 2022;22.

33. Exchange Rates UK. US Dollar to Sri Lankan Rupee Spot Exchange Rates for 2019 and 2020 [Internet]. USD LKR Historical Chart for Year 2019 & 2020. 2020 [cited 2022 Jul 12]. Available from: https://www.exchangerates.org.uk/USD-LKR-spot-exchange-rates-history-2020.html

34. Tusl M, Kerksieck P, Brauchli R, Bauer GF. Perceived impact of the COVID-19 crisis on work and private life and its association with mental well-being and self-rated health in German and Swiss employees: a cross-sectional study. BMC Public Health. 2021;21(741):1–21.

35. Rauf N, Zulfiqar S, Mumtaz S, Maryam H, Shoukat R, Malik A, et al. The impact of the covid-19 pandemic on pregnant women with perinatal anxiety symptoms in pakistan: A qualitative study. Int J Environ Res Public Health. 2021;18(16).

36. Shafi M, Liu J, Jian D, Rahman IU, Chen X. Impact of the COVID-19 pandemic on rural communities: A cross-sectional study in the Sichuan Province of China. BMJ Open. 2021;11(8).

37. Almeida V, Barrios S, Christl M, De Poli S, Tumino A, van der Wielen W. The impact of COVID-19 on households´ income in the EU. J Econ Inequal. 2021;19(3):413–31.

38. Chandra A, Sarda R, Kumar A, Bir M, Parija PP, Pal A, et al. Impact of COVID‑19 on the patients' income and work in Delhi, India. J Fam Med Prim Care [Internet]. 2021;10:3047–50. Available from: http://www.jfmpc.com/article.asp?issn=2249-4863;year=2017;volume=6;issue=1;spage=169;epage=170;aulast=Faizi

39. Celik B, Ozden K, Dane S. The Impact of COVID-19 on Household Economy and Consumption Preferences: An International Survey. J Glob Econ Bus [Internet]. 2020;01(02):91–115. Available from: http://www.journalsglobal.com/index.php/jgeb/article/view/29

40. Kansiime MK, Tambo JA, Mugambi I, Bundi M, Kara A, Owuor C. COVID-19 implications on household income and food security in Kenya and Uganda: Findings from a rapid assessment Monica K. Kansiime a,⇑, Justice A. Tambo b, Idah Mugambi

For peer review only

a, Mary Bundi a, Augustine Kara c, Charles Owuor d a CABI Africa, P.O. Box 633-00621. 2020;(January).

41. Syafiq A, Fikawati S, Gemily SC. Household food security during the COVID-19 pandemic in urban and semi-urban areas in Indonesia. J Heal Popul Nutr [Internet]. 2022;41(1):1–8. Available from: https://doi.org/10.1186/s41043-022-00285-y

42. Murakami E. Immediate impacts of the COVID-19 pandemic on household economic activities and food security in Tajikistan [Internet]. Vol. 6, Economics of Disasters and Climate Change. Springer International Publishing; 2022. 259–291 p. Available from: https://doi.org/10.1007/s41885-021-00104-4

43. Kittiprapas S. Socioeconomic impacts of the COVID-19 pandemic on the vulnerable households: empirical evidence from slum areas of Bangkok city. Cogent Soc Sci [Internet]. 2022;8(1). Available from: https://doi.org/10.1080/23311886.2022.2074111

44. Gunarathne S, Wickramasinghe N, Agampodi T, Prasanna I, Agampodi S. Nutritional Status and Additional Expenditure on Food During Pregnancy: Evidence From the Rajarata Pregnancy Cohort. Curr Dev Nutr [Internet]. 2021;5(2):754. Available from: https://academic.oup.com/cdn/article/5/Supplement_2/754/6293027

45. Gunarathne SP, Amarasinghe G, Agampodi TC, Wickramasinghe ND, Mendis V, Prasanna IR, et al. Does Being Diagnosed As Anemic in Early Pregnancy Have an Impact on the Household Expenditure for Food? Evidence From a Maternal Cohort in Rural Sri Lanka. Curr Dev Nutr [Internet]. 2022;6(1):654. Available from: https://academic.oup.com/cdn/article/6/Supplement_1/654/6606330

46. Gunarathne S, Wickramasinghe N, Agampodi T, Prasanna I, Agampodi S. Household Food Expenditure Share During Pregnancy: Evidence From Rural Sri Lanka. Curr Dev Nutr [Internet]. 2021;5(5):753. Available from: https://academic.oup.com/cdn/article/5/Supplement_2/753/6292904?login=false

47. Agampodi TC, Hromi-Fiedler A, Agampodi SB, Amarasinghe GS, Wickramasinghe ND, Jayasinghe IU, et al. A self-applied valid scale for rapid tracking of household food insecurity among pregnant women in Sri Lanka. Matern Child Nutr. 2021;17(3):1–7.

48. Padhye R, Purushotham A, Paul M, Sardeshpande N, Ballala R, Dhar S, et al.

For peer review only

Accessing Maternal Health Care in the Midst of the COVID-19 Pandemic: A Study in Two Districts of Assam, India. Front Glob Women's Heal. 2022;3(March):1–9.

49. Bailey V. Out-of-Pocket Healthcare Spending Increased Leading Up to Pandemic [Internet]. PRACTICE MANAGEMENT NEWS. 2022 [cited 2022 Oct 24]. Available from: https://revcycleintelligence.com/news/out-of-pocket-healthcare-spending-increased-leading-up-to-pandemic

50. Rabbani U, Saigul AA, Sulaiman A, Ibrahim TH. Impact of COVID-19 on Antenatal Care Utilization Among Pregnant Women in Qassim, Saudi Arabia. Cureus. 2021;13(11).

51. Banke-Thomas A, Semaan A, Amongin D, Babah O, Dioubate N, Kikula A, et al. A mixed-methods study of maternal health care utilisation in six referral hospitals in four sub-Saharan African countries before and during the COVID-19 pandemic. BMJ Glob Heal. 2022;7(2):1–14.

52. Sinha B, Dudeja N, Mazumder S, Kumar T, Adhikary P, Roy N, et al. Estimating the Impact of COVID-19 Pandemic Related Lockdown on Utilization of Maternal and Perinatal Health Services in an Urban Neighborhood in Delhi, India. Front Glob Women's Heal. 2022;3(March):1–10.

53. Muhaidat N, Fram K, Thekrallah F, Qatawneh A, Al-Btoush A. Pregnancy during COVID-19 outbreak: The impact of lockdown in a middle-income country on antenatal healthcare and wellbeing. Int J Womens Health. 2020;12:1065–73.

54. Agampodi TC, Rheinländer T, Agampodi SB, Glozier N, Siribaddana S. Social capital and health during pregnancy; An in-depth exploration from rural Sri Lanka. Reprod Health. 2017;14(1):1–19.

For peer review only

Supplementary Table 1: Socio-demographic and economic characteristics of the study sample

Characteristic		Delivered during the COVID-19 pandemic (n=807, 55.3%)	Delivered before the COVID-19 pandemic (n=653, 44.7%)	Statistically significant difference/ associations*
Age of the pregnant women [Mean (SD)] in years		27.8 (5.1)	28.5 (5.9)	U=187,609.00 p=0.330
Ethnicity [n (%)]	Sinhalese	542 (85.1)	699 (86.9)	X^2 [df=1]=1.022 p=0.312
	Other minority ethnic groups[1]	95 (14.9)	105 (13.1)	
Religion [n (%)]	Buddhist	542 (85.1)	685 (85.2)	X^2 [df=1]=0.004 p=0.952
	Other religions[2]	95 (14.9)	119 (14.8)	
Educational status [n (%)]	Primary education	4 (0.7)	9 (1.2)	X^2 [df=3]=3.221 p=0.359
	Junior secondary education	21 (3.5)	39 (5.0)	
	Senior secondary education	484 (80.0)	612 (79.2)	
	Higher education	96 (15.9)	113 (14.6)	
Daily wage group [n (%)]	Yes	130 (21.0)	98 (18.7)	X^2 [df=1]=0.909 p=0.340
	No	489 (79.0)	425 (81.3)	
Income-generating members in the household	Only one person	683 (80.6)	379 (81.3)	X^2 [df=1]=0.093 p=0.760
	More than one person	164 (19.4)	87 (18.7)	
Income status in relation to the poverty line [n (%)][3]	Households below the national poverty line	102 (11.5)	62 (7.0)	X^2 [df=1]=0.632 p=0.260
	Households below the lower-middle income countries' poverty line	98 (11.1)	56 (6.3)	X^2 [df=1]=0.488 p=0.214
	Households below the extreme poverty line	61 (6.9)	30 (3.0)	X^2 [df=1]=0.528 p=0.324
Per visit pregnancy expenditure during the first trimester (USD)	Mean (SD)	5.98 (7.77)	5.71 (7.50)	U=205,265.50 p=0.503
	Median (IQR)	4.05 (0.89-7.95)	3.59 (0.88-7.75)	

For peer review only

Per visit pregnancy expenditure during the second trimester (USD)	Mean (SD)	5.90 (6.99)	5.79 (6.90)	U=168,747.00 p=0.605
	Median (IQR)	3.50 (0.84-8.29)	3.48 (1.03-8.54)	

Note: [1]Tamil, Moor, Malays, and others, [2]Catholic/Christian, Hindu, Islam, and others, [3]Income status before COVID-19 pandemic, *The statistical significance difference/association of socio-demographic, economic, and health information was tested between women delivered during and before the COVID-19 pandemic, Mann-Whitney U test was used for continues variables, and Chi-square goodness of fit test was used for categorical variables

For peer review only

BMJ Open

Impact of COVID-19 Pandemic on Health Service Utilization and Household Economy of Pregnant and Postpartum Women: A Cross-Sectional Study from Rural Sri Lanka

Journal:	*BMJ Open*
Manuscript ID	bmjopen-2022-070214.R1
Article Type:	Original research
Date Submitted by the Author:	11-Apr-2023

Complete List of Authors:	Gunarathna, Sajan; Rajarata University of Sri Lanka Faculty of Medicine and Allied Sciences, Community Medicine<br>Wickramasinghe, Nuwan Darshana; Rajarata University of Sri Lanka Faculty of Medicine and Allied Sciences, Department of Community Medicine<br>Agampodi, Thilini; Rajarata University of Sri Lanka Faculty of Medicine and Allied Sciences, Department of Community Medicine<br>Prasanna, Indika Ruwan; Rajarata University of Sri Lanka, Department of Economics<br>Agampodi, Suneth; Rajarata University of Sri Lanka Faculty of Medicine and Allied Sciences, Department of Community Medicine

**Primary Subject Heading**:	Public health
Secondary Subject Heading:	Health economics, Health services research, Infectious diseases
Keywords:	COVID-19, Health economics < HEALTH SERVICES ADMINISTRATION & MANAGEMENT, Public health < INFECTIOUS DISEASES

SCHOLARONE™
Manuscripts

I, the Submitting Author has the right to grant and does grant on behalf of all authors of the Work (as defined in the below author licence), an exclusive licence and/or a non-exclusive licence for contributions from authors who are: i) UK Crown employees; ii) where BMJ has agreed a CC-BY licence shall apply, and/or iii) in accordance with the terms applicable for US Federal Government officers or employees acting as part of their official duties; on a worldwide, perpetual, irrevocable, royalty-free basis to BMJ Publishing Group Ltd ("BMJ") its licensees and where the relevant Journal is co-owned by BMJ to the co-owners of the Journal, to publish the Work in this journal and any other BMJ products and to exploit all rights, as set out in our licence.

The Submitting Author accepts and understands that any supply made under these terms is made by BMJ to the Submitting Author unless you are acting as an employee on behalf of your employer or a postgraduate student of an affiliated institution which is paying any applicable article publishing charge ("APC") for Open Access articles. Where the Submitting Author wishes to make the Work available on an Open Access basis (and intends to pay the relevant APC), the terms of reuse of such Open Access shall be governed by a Creative Commons licence – details of these licences and which Creative Commons licence will apply to this Work are set out in our licence referred to above.

Other than as permitted in any relevant BMJ Author's Self Archiving Policies, I confirm this Work has not been accepted for publication elsewhere, is not being considered for publication elsewhere and does not duplicate material already published. I confirm all authors consent to publication of this Work and authorise the granting of this licence.

For peer review only

Title Page

Title

Impact of COVID-19 Pandemic on Health Service Utilization and Household Economy of Pregnant and Postpartum Women: A Cross-Sectional Study from Rural Sri Lanka

Details of the corresponding author

Full Name: Sajan Praveena Gunarathna

Postal address: Department of Community Medicine, Faculty of Medicine and Allied Sciences, Rajarata University of Sri Lanka, Saliyapura - Sri Lanka

Email**:** sajaanpraveen7@gmail.com

Names and affiliations of the authors

1. Sajan Praveena Gunarathna

Department of Community Medicine,

Faculty of Medicine and Allied Sciences,

Rajarata University of Sri Lanka,

Saliyapura - Sri Lanka.

2. Nuwan Darshana Wickramasinghe

Department of Community Medicine,

Faculty of Medicine and Allied Sciences,

Rajarata University of Sri Lanka,

Saliyapura - Sri Lanka.

3. Thilini Chanchala Agampodi

Department of Community Medicine,

Faculty of Medicine and Allied Sciences,

For peer review only

Rajarata University of Sri Lanka,
Saliyapura - Sri Lanka.

4. Indika Ruwan Prasanna
Department of Economics,
Faculty of Social Sciences and Humanities,
Rajarata University of Sri Lanka,
Mihintale – Sri Lanka.

5. Suneth Buddhika Agampodi
Department of Community Medicine,
Faculty of Medicine and Allied Sciences,
Rajarata University of Sri Lanka,
Saliyapura - Sri Lanka.

Word Count: 3,900 [excluding title page, abstract, strength and limitations, key messages, statements (Contributorship, Funding, Data sharing statements, Ethical approval, Competing Interests, and Acknowledgments), references, and tables]

For peer review only

Impact of COVID-19 Pandemic on Health Service Utilization and Household Economy of Pregnant and Postpartum Women: A Cross-Sectional Study from Rural Sri Lanka

Abstract

Objectives: This study aims to describe how household economies and health service utilization of pregnant and postpartum women were affected during the pandemic.

Design: A cross-sectional study.

Setting: This study was conducted in the Anuradhapura district, Sri Lanka.

Participants: The study participants were 1,460 pregnant and postpartum women recruited for the Rajarata Pregnancy Cohort (RaPCo) during the initial stage of the COVID-19 pandemic.

Primary and secondary outcome measures: Household economic (income, poverty, nutritional, and health expenditures) and health service utilization details during the COVID-19 pandemic were gathered through telephone interviews. Socio-demographic and economic data were obtained from the cohort baseline and analyzed with descriptive and non-parametric analysis.

Results: Out of the 1,460 women in the sample, 55.3% (n=807) were pregnant, and 44.7% (n=653) were postpartum women. Of the total sample, 1,172 (80.3%) women participated in the economic component. The monthly household income [median (IQR) = 212.39 (159.29-265.49)] reduced [median (IQR) = 159.29 (106.20-212.39)] in 50.5% (n=592) families during the pandemic ($Z=-8.555$, $p<0.001$). Only 10.3% (n=61) of affected families had received financial assistance from the government, which was only 46.4% of the affected income. The nutritional expenditure of pregnant women was reduced ($Z=-2.023$, $p=0.043$) by 6.7%. During the pandemic, 103 (8.8%) families with pregnant or postpartum women were pushed into poverty, and families who were pushed into poverty did not receive any financial assistance. The majority of women (n=1,096, 83.3%) were satisfied with the free public health services provided by the public health midwife during the pandemic.

For peer review only - http://bmjopen.bmj.com/site/about/guidelines.xhtml

Conclusion: During the early stages of the pandemic, healthcare utilization of pregnant women was minimally affected. Even before the country's current economic crisis, the household economies of pregnant women in rural Sri Lanka were severely affected, pushing families into poverty due to the pandemic. The impact of COVID-19 and the aftermath on pregnant women will have many consequences if the policies and strategies are not revised to address this issue.

Keywords: COVID-19 pandemic, Health services, Poverty, Pregnant women

Strengths and limitations of the study

- We conducted telephone interviews during the COVID-19 pandemic with a large sample of pregnant and postpartum women who were participants in a maternal cohort in Sri Lanka.
- We combined three large data sets (baseline and follow-up economic data and telephone interview data of the Rajarata Pregnancy Cohort study) to identify the changes in the household economy and health service utilization with the pandemic.
- The data quality and validity may be compromised due to the selection bias and the mode of interviews (telephone interviews), even though remote data collection is the alternative option available during a pandemic.
- Although we identified that the socio-demographic characteristics are not statistically significant different between pregnant and postpartum women in the present sample, the two groups may be qualitatively different due to non-response during the pandemic.

Key Messages

- The COVID-19 pandemic disrupted health service delivery and adversely affected the household economies, where pregnant and postpartum women were recognized as a vulnerable group.
- The household economy of pregnant women in Sri Lanka was severely affected during the pandemic pushing families into poverty.
- Pregnancy care service utilization was minimally affected in rural Sri Lanka in the initial phase of the pandemic, and a notable dedication of the field healthcare team to provide uninterrupted service was evident.

For peer review only

- A proper mechanism is needed to support and distribute government-led or other financial assistance for affected families with pregnant women, especially considering the economic crisis as an aftermath of the pandemic.

Introduction

Pregnant and postpartum women in low and middle-income countries are among the most vulnerable groups considering the indirect impact estimates due to health system collapse and food insecurity during the COVID-19 pandemic (1,2). Disrupted healthcare service delivery, accessibility, and poor healthcare utilization during the COVID-19 pandemic and the household economic crisis that followed in the aftermath are impediments to achieving Sustainable Development Goals (SDG) targets (3,4). The pandemic was an unexpected catastrophe in the global and national sociocultural and economic landscape affecting the daily well-being of households directly or indirectly (5,6). Many countries implemented stringent pandemic control measures, including national or local lockdowns (7), which led household economies into a crisis and affected health service utilization (8).

Many countries recommended spacing clinic visits during pregnancy and following up remotely by phone; only reserving emergency care was provided at the hospitals (9). Both high and low-income countries reported changes/reductions in maternal healthcare services during the COVID-19 pandemic. According to the available evidence, maternity care and birth practices changed in the United States of America, increased the number of home births, conducted prenatal visits through online platforms, and partners and support persons were excluded from birthing rooms leaving mothers unsupported (10). Nepal reported a reduction of institutional childbirth by more than half, with rises in neonatal mortality and poor quality of care (7). There has been a reduction in health service utilization in North-East Ethiopia due to fear of pregnant women attending health institutions (5). In addition, poor access to healthcare care and reduced perceived social support for pregnant women were reported around the globe (11). Thus, the COVID-19 pandemic has adversely affected the utilization of maternal healthcare services.

Few studies have cited the adverse impact of the COVID-19 pandemic on the household economy (6,8). These studies investigated the adverse effects on household economies in Nigeria (12), changes in consumption patterns of Malaysian households (13), losing jobs and declining household income in some Asia-pacific countries—Cambodia, the Lao People's

For peer review only

For peer review only - http://bmjopen.bmj.com/site/about/guidelines.xhtml

Democratic Republic, Malaysia, Indonesia, the Philippines, Myanmar, Thailand, and Vietnam (14). Economic burden during COVID-19 could be more problematic for households of pregnant women since pregnancy expenditure was already a burden in many low and middle-income countries (15). However, studies explicitly focusing on the COVID-19 impact on the household economy of pregnant women are lacking in the global evidence base.

Sri Lanka is a lower-middle-income country (16) where maternal health services are well developed and accessed by pregnant women via both the public (to a more significant extent) and the private health system (17,18). The government's free public health system delivers maternal care through a well-established network of primary healthcare officers, reporting 85.3% of pregnant women registered in recent years (19). Also, the country is among the leads of lower-middle-income countries regarding maternal and child health SDGs. Sri Lanka has reached the fourth phase of the obstetric transition (20), and the healthcare system of Sri Lanka is an excellent example of universal public health system coverage, disease prevention through evidence-based strategies, and using culturally appropriate manners for health promotion, etc. (18). The solid network of public health personnel, especially the public health midwives (PHMs) that involve in domiciliary and clinic care in the field has been an asset to the system.

The first local index case emerged on 11th March 2020, and lockdowns/curfews were initiated after 17th March 2020 (21). During the COVID-19 pandemic's initial stage, a low mortality rate due to COVID-19 was recorded with the high public health system standards in Sri Lanka (21). The interim guidelines on maternal healthcare services were provided to sustain the services during the pandemic by the Family Health Bureau, Ministry of Health, the national focal point of maternal healthcare in the Ministry of Health, Sri Lanka (22,23). It provided expert guidance on the procedures for organizing services, home visits, clinic services, communications, and protective measures (22). However, the COVID-19 pandemic caused a higher prevalence of anxiety and depression among pregnant women, requiring additional support from the family and field health officers (24). This could be worse if the household economy were affected since the income status was associated with the pregnancy expenditure (25,26) and pregnancy nutritional status (27). Available evidence indicates that the COVID-19 pandemic created an economic meltdown at the household level and the Sri Lankan economy (28).

For peer review only - http://bmjopen.bmj.com/site/about/guidelines.xhtml

How many Sri Lankan maternal health services have been affected by the poor economic conditions of the household during the COVID-19 pandemic is not well explored. The empirical studies explicitly focusing on the impact of the COVID-19 pandemic on maternal healthcare service utilization and the household economy of pregnant women in Sri Lanka are lacking. Assessing the effects of the COVID-19 epidemic on the household economy and health service utilization of pregnant and postpartum women would yield valuable evidence for decision-making during pandemic situations. Thus, this study aimed to find whether the COVID-19 pandemic affected the household economy and health service utilization of pregnant and postpartum women in rural Sri Lanka.

Methods

Study Design and study setting

We conducted a cross-sectional study via telephone interviews. The participants were the women in the economic component of the largest pregnancy cohort in Sri Lanka, the Rajarata Pregnancy Cohort (RaPCo) (29, 30). Anuradhapura district is the largest district in Sri Lanka (7,179 km^2), with a current population of about 900,000, and most of the population (92.7%) belongs to the district's rural population. The agriculture industry is the primary source of income, and the median monthly household income is USD 285.91 (31). Total health expenditure comprises 58% of government sources (32), and maternal, postpartum, and child health care is provided free of charge by the government sector through 22 Medical Officer of Health (MOH) areas and 275 Public Health Midwife (PHM) areas. Approximately 17,000 pregnant women register at the PHM annually, and the number of live births is about 15,000 (29). Further details on the study setting have already been published (29).

Study population and sample

The study population included pregnant women registered in the Anuradhapura district's maternal care program, where the antenatal care coverage is 82.3% (29). All pregnant women fulfilling the inclusion criteria were invited for the baseline assessment of the RaPCo study from 01 July 2019 to 30 September 2019 to participate in this study. The eligibility criteria of the RaPCo were as follows:

For peer review only - http://bmjopen.bmj.com/site/about/guidelines.xhtml

- Pregnant women registered at the PHM and visiting antenatal field clinics in the Anuradhapura district
- Pregnant women whose permanent residence is in the Anuradhapura district and had not planned to leave the district until childbirth
- Period of amenorrhoea/gestational age is less than 13 weeks by the time of recruitment

Pregnant women with uncertain due dates and those who planned to leave the study setting for delivery were excluded from the study. The pregnant women for the study were recruited with the help of PHMs in each MOH area by organizing a special clinic with the permission of the district's public health authorities. Informed written consent was sought from the participants before the study, and ethical approval and administrative clearance were obtained before the recruitment. The original cohort included >90% of the study population. All pregnant women recruited were invited for the baseline economic study.

Data collection

With the permission of pregnant women during recruitment of the RaPCo, we collected their telephone numbers to contact them to share the investigation results and follow-up data collection. We held a telephone interview using a pre-tested interviewer guide with close-ended questions and collected details on the routine utilization of antenatal/postnatal health services and household financial information during the pandemic (33). Data collection was based on five major categories: 1) whether income was affected during the outbreak, 2) financial assistance received, 3) the status of utilizing maternal health services, 4) the assistance of the PHM, and 5) the assistance of household members/neighbors (Supplementary File 1).

Five female medical graduates trained in telephone interview techniques, emphasizing politeness, telephone etiquette, basic counseling skills, and essential perinatal health information, carried out the interviews. If the first attempt failed, several attempts were made to contact pregnant women on the same day and subsequent days for the next two weeks. The second round of calls was done to reach those unavailable or busy the first time. Telephone interviews were conducted mainly during the pandemic and completed within three months (from April to June 2020). The duration of the interview was 4-10 minutes.

We explained the study objective and confirmed whether the study participation was voluntary. We obtained verbal consent during the interview apart from the written consent

For peer review only - http://bmjopen.bmj.com/site/about/guidelines.xhtml

sorted at the recruitment in the RaPCo study. During the telephone interviews, we provided psychological support to pregnant and postpartum women where needed.

Definition of measures

We presented the sample characteristics and third trimester per visit pregnancy expenditure between women delivered during (after 17 March 2020) and before (before 17 March 2020) the COVID-19 pandemic. We considered 17 March 2020 as the cut-off date to create this variable since the national lockdowns/curfews began on 17 March 2020 (21).

To identify the impact of the COVID-19 pandemic on the household economy, we used monthly household income, poverty status, and financial assistance during the pandemic as the main variables. Monthly household income was the total monthly earnings of all household members during the pandemic. The national (USD 3.65) and extreme (USD 2.15) poverty lines were used for poverty analysis. For the status of receiving financial assistance, we considered only the government financial assistance (value of USD 26.55). It was the only financial aid provided by the government, provided monthly for three months and only for selected families living under the national poverty line.

Furthermore, we created a few variables, i.e.,

- Income reduction of ith household = Income before COVID-19 pandemic of the ith household – Income during COVID-19 pandemic of the ith household.
- Percentage of affected income over household income of the ith household = (Reduced income of the ith household × 100%) / Household income of the ith household.

The same formula was used to create the percentage of received financial aid over affected income, the percentage of received financial aid over household income, and the percentage of received financial aid over household expenditure.

Testing the differences in per-visit pregnancy expenditure during the third trimester was made based on the similarity of socio-demographic profiles within two groups (Supplementary Table 1), and all pregnant women who delivered during COVID-19 were in their third trimester. Moreover, the per month pregnancy-related nutritional expenditure was the amount spent for pregnant women’s dietary intake per month.

For peer review only - http://bmjopen.bmj.com/site/about/guidelines.xhtml

To identify the status of health service utilization during the pandemic, we used the following variables: missed clinics, number of missing clinic visits, whether changed the mode and place of childbirth and home visits of PHM, etc. Also, to measure the status of support received from the PHM and the family members, we used the Likert scale, where one denoted ‘strongly dissatisfied’ and five denoted ‘strongly satisfied.’

Data analysis

We entered data during the interviews using Microsoft Office Excel software and data management using the same. Next, we combined the baseline data collected from the RaPCo and the economic study with this database and analyzed data using SPSS V27. Before the analysis, all monetary values were converted to the average exchange rate (USD to Sri Lankan Rupees [LKR]) of the data collection period from April 2020 to June 2020 (i.e., USD 1 = LKR 188.33) (34). The average exchange rate for the above period was based on the fact that there were no outliers, and the standard deviation was only USD 2.99.

Sample characteristics (including socio-demographic, economic, and health-related information), health service utilization information, and household financial information during the pandemic were presented using univariate descriptive statistical measures. Since the data were not normally distributed ($p<0.05$) due to the Shapiro-Wilk test, we used non-parametric statistical tests for bivariate analysis, including the Wilcoxon Signed Ranks Mann-Whitney U test and Chi-square goodness of fit test.

Patient and public involvement

The telephone follow-up interviews for the RaPCo study were developed based on community engagement. Pregnant women recruited in RaPCo called the 24-hour helpline during the COVID-19 lockdown, expressing concerns about their health status, psychological issues and accessing help. The research team took note of these issues raised by the participants to conceptualize the telephone-based follow-up study focusing on financial, social, and health problems and offered psychological support as well as appropriate interventions. The questions for the calls were also based on inquiries from the participants. While the participants were not directly involved in the design, their feedback and engagement influenced the development of the follow-up method.

Results

For peer review only - http://bmjopen.bmj.com/site/about/guidelines.xhtml

We invited all pregnant women of the RaPCo (n=3,374), and 1,460 women participated in this study (response rate = 43.3%). This was the only number contactable during the pandemic, and no woman opted out during the interview. The household income (p=0.743), household expenditure (p=0.512), religion (p=0.363), ethnicity (p=0.515), education level (p=0.361), and status of sexual and reproductive health education (p=0. 344) were not significantly different between the present study sample participants and other RaPCo participants.

Sample characteristics

The mean (SD) age of the pregnant and postpartum women was 28.2 (5.5) years. Among the total sample (n=1,460), 44.7% (n=653) of women delivered before the COVID-19 pandemic and 55.3% (n=807) during the COVID-19 pandemic. Table 1 shows the socio-demographic and economic characteristics of the study sample.

Table 1: Socio-demographic and economic characteristics of the study sample

Characteristic		Statistics (n=1,460, 100%)
Age of the pregnant women [Mean (SD)] in years		28.2 (5.5)
Ethnicity [n (%)]	Sinhalese	1,250 (85.6)
	Other minority ethnic groups[1]	210 (14.4)
Religion [n (%)]	Buddhist	1,237(84.7)
	Other religions[2]	223 (15.3)
Educational status [n (%)]	Primary education	15 (1.0)
	Junior secondary education	63 (4.3)
	Senior secondary education	1,162 (79.6)
	Higher education	220 (15.1)
Daily wage group [n (%)]	Yes	296 (20.3)
	No	1,164 (79.7)
Income-generating members in the household	Only one person	1,180 (80.8)
	More than one person	280 (19.2)
Income status in relation to the poverty line [n (%)][3]	Households below the national poverty line	173 (11.8)
	Households below the extreme poverty line	112 (7.8)
Per visit pregnancy expenditure during the first trimester (USD) [median (IQR)]		3.72 (0.88-7.88)

For peer review only

For peer review only - http://bmjopen.bmj.com/site/about/guidelines.xhtml

Per visit pregnancy expenditure during the second trimester (USD) [median (IQR)]	3.45 (0.88-8.50)

Note: [1]Tamil, Moor, Malays, and others, [2]Catholic/Christian, Hindu, Islam, and others, [3]Income status before COVID-19 pandemic

Impact of COVID-19 Pandemic on household economy

The pregnant and postpartum women's monthly household income showed a statistically significant difference (p<0.001) during the pandemic and the pre-COVID period. The household income was affected in 50.5% (n=592) of families, and the percentage of reduced income in terms of household income was 74.6%. Though the monthly household income was reduced, there was no statistically significant difference (Z=-0.827, p=0.408) in household expenditure during and before the COVID-19 pandemic for the families of women who delivered during COVID-19 (n=807, 55.3%).

The majority (n=1,111, 94.8%) had not received government financial assistance, and the obtained amount covered only 46.4% of the affected income (Table 2).

Table 2: COVID-19 impact on the household economy, pregnancy expenditure, and status of receiving financial assistance

Description			Statistics [n=1,172, 100%]
Impact on household income	Families affected by income reduction [n (%)]	Yes	592 (50.5)
		No	580 (49.5)
	Reduced amount (USD)	n (%)	592 (50.5)
		Median (IQR)	106.20 (53.10-159.29)
	Percentage of reduced income over household income (%)		74.6
Monthly household income (USD) changes	n (%)		592 (50.5)
	Income before COVID-19 [median (IQR)]		212.39 (159.29-265.49)
	Income during COVID-19 [median (IQR)]		159.29 (106.20-212.39)
	Statistically significant difference[1]		p<0.001
Households pushed into poverty [n (%)]	National poverty line		56 (4.8)
	Extreme poverty line		47 (4.0)
Financial assistance provided by the	Received financial aid among families pushed into poverty [n (%)]		2 (1.9)

For peer review only

For peer review only - http://bmjopen.bmj.com/site/about/guidelines.xhtml

government (USD 26.55)	Received financial assistance among affected families [n (%)]	61 (10.3)
	Percentage of received financial aid over affected income (%)	46.4
	Percentage of received financial aid over household income (%)	17.9
	Percentage of household expenditure covered by the received financial aid (%)	29.9
Per month pregnancy-related nutritional expenditure (n=807, 55.3%)	Before the COVID-19 period [median (IQR)]	USD 54.43 (37.17-79.65)
	During the COVID-19 period [median (IQR)]	USD 53.10 (37.17-79.65)
	Statistically significant difference[1]	p=0.043
Per visit out-of-pocket expenditure in the third trimester	Delivered before COVID-19 Pandemic[2] [n=524, 44.7%] [median (IQR)]	4.82 (0.73-10.19)
	Delivered during COVID-19 Pandemic[3] [n=648, 55.4%] [median (IQR)]	4.73 (1.02-9.64)
	Statistically significant difference[4]	p=0.771

Note: [1]Wilcoxon signed ranks test, [2]The cost indicated here was incurred before the COVID-19 pandemic, [3]The cost indicated here was incurred during the COVID-19 pandemic.

Daily wage earners' (n=296, 20.3%) income was reduced by 80.0%. Only 14.5% (n=43) of families in the daily wage group received government financial assistance (USD 26.55), covering 57.6% of affected income, 19.1% of household income, and 33.5% of household expenditure.

Among the total sample, a considerable number of families (n=103, 8.8%) were pushed into poverty and extreme poverty due to the pandemic. The total affected amount [median (IQR) = USD 106.20 (53.10-159.29)] was equivalent to 72.7% of their monthly household income. However, only 10.3% (n=61) of affected families had received financial assistance from the government, which was 54.3% of the affected income, 31.0% of the monthly household income, and 40.4% of the monthly household expenditure. Financial assistance was not given to families with pregnant women who were pushed into poverty; in total, only 1.9% (n=2) of families who were pushed into poverty received financial assistance. The breakdown analysis of the impact of the household economy between pregnant (delivered during COVID-19) and postpartum women (delivered before COVID-19) was reported in Supplementary Table 2.

For peer review only

For peer review only - http://bmjopen.bmj.com/site/about/guidelines.xhtml

A statistically significant difference (Z=-2.020, p=0.043) was noted in per-month pregnancy-related nutritional expenditure during and before the COVID-19 pandemic. The reduction in nutritional spending was 6.7% compared with early pregnancy. However, there was not a statistically significant difference (U=293.000, p=0.582) of pregnancy-related nutritional expenditure during COVID-19 outbreak between families which faced income loss and their counterparts.

There was no statistically significant difference (U=19,668.00, p=0.771) in spending per visit during COVID-19 compared to pre-COVID-19. An extended analysis of pregnancy expenditure breakdown [Direct medical (cost for consultation, medicine, laboratory investigation) and non-medical pregnancy expenditure (cost for traveling, food and refreshments, accompanying person)] was reported in Supplementary Table 3.

Impact of COVID-19 on routine health service utilization of Pregnant and postpartum women

Of the total sample, 14.2% (n=104) of pregnant women and 6.9% (n=40) of postpartum women reported missing clinics due to the COVID-19 pandemic. The median (IQR) number of missed clinics was 1 (1-2). The majority (n=1,096, 83.3%) of the pregnant and postpartum women were satisfied with the service provided by the PHM during the pandemic. Most women (n=1,170, 89.0%) visited/received PHM phone check-ups, and the median (IQR) visits/phone check-ups were 2 (1-3). Furthermore, most pregnant (n=1,092, 83.1%) and postpartum women were satisfied with the support/assistance received by their spouse/family members and neighbors during the COVID-19 pandemic. Only 0.5% (n=4) of pregnant women had to change the childbirth mode from normal vaginal delivery to lower segmant C-section, and 1.9% (n=14) women had to change the place of childbirth due to the travel restrictions of the curfew period (Table 3).

There is no association between the health service utilization of pregnant women and the income changes during the COVID-19 pandemic [$X^2(df=1)=0.361, p=0.548$]. The breakdown analysis of the impact of health service utilization between pregnant (delivered during COVID-19) and postpartum women (delivered before COVID-19) was reported in Supplementary Table 4.

Table 3: Impact of COVID-19 on health service utilization of pregnant and postpartum women and support received by PHM and family

For peer review only - http://bmjopen.bmj.com/site/about/guidelines.xhtml

Description			Statistics [n=1,315, 100%]
Clinic visits[1]	Whether missed clinics [n (%)]	Yes	144 (11.0)
		No	1,171 (89.0)
	Number of missed clinics [median (IQR)]		1 (1-2)
Field health services from PHM	Whether PHM visited or did phone check-ups to see the pregnant women/infant [n (%)]	Yes	1,170 (89.0)
		No	145 (11.0)
	Number of visits per month [median (IQR)]		2 (1-3)
	Service provided by the PHM [n (%)]	Strongly satisfied	7 (0.5)
		Satisfied	1,096 (83.3)
		Average	188 (14.3)
		Not satisfied	18 (1.4)
		Strongly not satisfied	6 (0.5)
Childbirth	Whether changed the mode of childbirth [n (%)]	Yes	4 (0.5)*
		No	728 (99.5)
	Whether changing the place of childbirth [n (%)]	Yes	14 (1.9)**
		No	718 (98.1)
Support from husband/family and neighbors received during the pandemic [n (%)]	Very satisfied		16 (1.2)
	Satisfied		1,092 (83.1)
	Average		161 (12.2)
	Dissatisfied		27 (2.1)
	Very dissatisfied		19 (1.4)

Note: [1]Indicated clinic visits are antenatal clinic visits for the women delivered during COVID-19 and postnatal/child health clinics for women delivered before COVID-19, *Number of pregnant women expected to change the MOD, **Number of pregnant women expected to change the place of childbirth

Discussion

This study assessed the COVID-19 pandemic impact on health service utilization and the household economy of pregnant and postpartum women in rural Sri Lanka. Since the study was based on the follow-up of a maternal cohort, we could compare the impact on the household economy with the pre-COVID values. We found that the household income is reduced by 74.6% in most families. Totally, 103 families (8.8%) were pushed below the

For peer review only - http://bmjopen.bmj.com/site/about/guidelines.xhtml

poverty line, while 19.5% of families already remained in the poorest group. We identified that the most vulnerable group was daily income earners, and the financial aid received was two fifth-a small share compared with the income loss.

No changes were observed in per-visit pregnancy expenditure between pregnant women who delivered during and before the COVID-19 period. However, minor differences were noted in the cost of medicine, the cost for accompanying persons, and direct medical expenditures. We also detected a reduction in the nutritional expenditure of pregnant women during the COVID-19 period. Health services were minimally affected, and health service utilization was not associated with income reduction. Further, pregnant and postpartum women were highly satisfied with the services provided by PHM.

Impact on household economy and pregnancy expenditure

Even though it is the early stage of the COVID-19 pandemic, most households' income was affected by the local and national pandemics. Almost half of the families experienced an income reduction; the reduced income was equivalent to three-quarters of the monthly household income. The significant number of families pushed into poverty (n=56, 4.8%) and extreme poverty (n=47, 4.0%) show the pandemic's severity. The most vulnerable group was daily income earners, who were one-fifth of the total sample. The reason was unavailability and the inability to seek work due to pandemics and curfews (35). Daily income earners must spend 70.0% of per day's income per visit to antenatal care in rural Sri Lanka (26). As reported in Pakistan (36), this could impact pregnant women's mental health. Household income reduction and poverty due to the pandemic were common phenomena, as documented by many researchers (37-39) .

Food insecurity and changes in food consumption patterns due to income reduction during the COVID-19 pandemic were recorded in Kenya and Uganda (40), Indonesia (41), Tajikistan (42), Thailand (43), and in multi-country surveys (44). Although nutritional status is considered necessary in pregnancy, nutritional expenditure is reduced compared to early pregnancy due to income reduction. This could be a sensitive matter since 16.5% of pregnant women were recorded as underweight and 14.5% as anemic in the sample, as reported in other studies conducted in the same study setting (45–47). Household food diversity and nutritious food intake are compromised at the early stage of food insecurity in the same population of pregnant women even before the pandemic (48). Hence, necessary policies and

For peer review only - http://bmjopen.bmj.com/site/about/guidelines.xhtml

mechanisms must be implemented to safeguard the food security of pregnant women at-risk during similar situations.

The burden of out-of-pocket expenditure has been highlighted despite accessing free services from government health facilities in India during the COVID-19 pandemic (49). However, with the organized and freely provided government maternal health care, the pregnancy expenditure was expected to be at a lower level compared to the pre-COVID-19 period since the pregnancy out-of-pocket expenditure showed a global decline of 16.2% (50).

In this study, the pregnancy expenditure of the pregnant women delivered during the COVID-19 pandemic did not differ from the expenditure incurred for pregnant women delivered before the COVID-19 pandemic. Pregnancy expenditure is identified as an economic burden in the same study setting since it exceeds more than 10% of household income (25,26). This is problematic for the households whose income was affected, pushed into poverty, and the pregnancy and household expenditure remained unchanged during the pandemic.

Although the Sri Lankan government provided low-income people with financial assistance (USD 26.55), pregnant women's families who were pushed into poverty did not receive financial aid, and only 1.9% of families who were pushed into poverty received financial assistance. However, only 10.3% of the affected families received it, while 19.5% were already below the poverty line, adding more than 8.8% to poverty due to the pandemic. The received financial aid was a small share compared to income loss and household expenditures during the COVID-19 pandemic.

Health services during the pandemic

During the COVID-19 pandemic, missing antenatal clinic care was reported in Saudi Arabia (51), Sub-Saharan Africa (52), India (53), Pakistan (36), and almost all middle-income countries (54). Similarly, in Sri Lanka, all routine healthcare services were interrupted due to lockdown, police curfew, and travel restrictions (21). Our study shows that the pandemic has resulted in missed antenatal clinics, yet the pregnant women were highly satisfied with the service provided by the field healthcare staff. In addition, support received from the spouses/family and neighbors was reported as 'satisfactory.' These results differ from other studies where reduced perceived family/social support was reported in the United States of America, Ireland, and the United Kingdom (11).

For peer review only

For peer review only - http://bmjopen.bmj.com/site/about/guidelines.xhtml

Sri Lanka's efficient public health system delivers maternal care through a well-established network of field staff and primary health care officers. The system provides domiciliary care and clinic-based service through grass-root level health officers, mainly via PHMs (29). During the COVID-19 pandemic, health authorities in Sri Lanka provided interim guidelines highlighting that the PHM needs to do domiciliary visits to the infection control procedures and contact over the phone (22,23). Our study shows that implementing those guidelines is up to the level of satisfaction of the recipients during the very early part of the pandemic (April-June 2020). The identified 'no association' between the impact of COVID-19 on the household economy and health service utilization could also be partly attributed to the free public health system. Also, the available evidence of the region reported that families of pregnant women compromise other expenditures regarding pregnancy-related matters, and even low-income families save money for pregnancy needs (55).

Conclusions

During the early stage of the COVID-19 pandemic in rural Sri Lanka, the healthcare utilization of pregnant women was minimally affected, despite the adverse impact on the household economy and healthcare delivery. However, the economic consequences pushed families into poverty, reducing nutritional expenditure. Financial aid was insufficient and not received by all in need. The post-pandemic economic crisis may pose a significant threat to the Sri Lankan maternal health system, requiring targeted and strategic government and international aid. This paper highlights only the immediate impact and calls for further studies to assess the long-term effects of the pandemic.

Contributorship statement: SPG, NDW, and SBA were responsible for the study concept and design. SPG and TCA were involved in data and project management. SPG performed data cleaning and analysis, drafted and interpreted the data, and drafted the manuscript. NDW, IRP, TCA, and SBA reviewed the manuscript. SPG is responsible for the overall content as the guarantor. All authors approved the final manuscript as submitted and agree to be accountable for all aspects of the work.

Competing Interests: The authors declare that they have no competing interests.

Funding: The Accelerating Higher Education Expansion and Development (AHEAD) Operation of the Ministry of Higher Education, Sri Lanka, which was funded by the World Bank, supported this research. The funding agency has no role in the study's design,

For peer review only

For peer review only - http://bmjopen.bmj.com/site/about/guidelines.xhtml

collection, analysis, interpretation of data, and manuscript writing. The grant number is DOR STEM HEMS [6026-LK/ 8743-LK].

Data sharing statement: Data of the present study are from the RaPCo study and are not publicly available. The datasets used during the current study are available from the corresponding author upon reasonable request.

Ethical approval: This study was conducted under the large cohort study in Anuradhapura District, Sri Lanka, The Rajarata Pregnancy Cohort (RaPCo). Ethical clearance for the RaPCo study was obtained from the "Ethics Review Committee of the Faculty of Medicine and Allied Sciences, Rajarata University of Sri Lanka (Reference no: ERC/2019/07)". Under the Declaration of Helsinki, all participants were informed that this study was voluntary and that data handling would be confidential. All participants gave their informed verbal consent.

Acknowledgments: We acknowledge Dr. Imasha Jayasinghe, Department of Community Medicine, Faculty of Medicine and Allied Sciences, Rajarata University of Sri Lanka, for the extensive support during the data collection.

References

[1]. Roberton T, Carter ED, Chou VB, Stegmuller AR, Jackson BD, Tam Y, et al. Early estimates of the indirect effects of the COVID-19 pandemic on maternal and child mortality in low-income and middle-income countries: a modelling study. Lancet Glob Heal [Internet]. 2020;8(7):e901–8. Available from: http://dx.doi.org/10.1016/S2214-109X(20)30229-1

[2]. World Health Organization (WHO). Coronavirus disease (COVID-19): Pregnancy and childbirth. 2020.

[3]. Kisiangani I, Elmi M, Bakibinga P, Mohamed SF, Kisia L, Kibe PM, et al. Persistent barriers to the use of maternal, newborn and child health services in Garissa sub-county, Kenya: A qualitative study. BMC Pregnancy Childbirth. 2020;20(1):1–12.

[4]. Akaba G, Dirisu O, Okunade K, Adams E, Ohioghame J, Obikeze O, et al. Impact of COVID-19 on utilization of maternal, newborn and child health services in Nigeria: protocol for a country-level mixed-methods study. F1000Research. 2020;9:1106.

[5]. Assefa KT, Gashu AW, Mulualem TD. The impact of COVID-19 infection on

For peer review only - http://bmjopen.bmj.com/site/about/guidelines.xhtml

maternal and reproductive health care services in governmental health institutions of Dessie town, North-East Ethiopia, 2020 G.C. medRxiv. 2020;2020.09.20.20198259.

[6]. Menendez, Clara; Gonzalez, Raquel ; Donnay F, Leke RGF. Avoiding indirect effects of COVID-19 on maternal and child health. Lancet Glob Heal. 2020;(January):19–21.

[7]. KC A, Gurung R, Kinney M V., Sunny AK, Moinuddin M, Basnet O, et al. Effect of the COVID-19 pandemic response on intrapartum care, stillbirth, and neonatal mortality outcomes in Nepal: a prospective observational study. Lancet Glob Heal. 2020;8(10):e1273–81.

[8]. Martin A, Markhvida M, Hallegatte S, Walsh B. Socio-Economic Impacts of COVID-19 on Household Consumption and Poverty. Econ Disasters Clim Chang. 2020;4(3):453–79.

[9]. Abdelbadee AY, Abbas AM. Impact of COVID-19 on reproductive health and maternity services in low resource countries. Eur J Contracept Reprod Heal Care. 2020;25(5):402–4.

[10]. Davis-Floyd R, Gutschow K, Schwartz DA. Pregnancy, Birth and the COVID-19 Pandemic in the United States. Med Anthropol Cross Cult Stud Heal Illn. 2020;39(5):413–27.

[11]. Meaney S, Leitao S, Olander EK, Pope J, Matvienko-Sikar K. The impact of COVID-19 on pregnant women's experiences and perceptions of antenatal maternity care, social support, and stress-reduction strategies. Women and Birth [Internet]. 2022;35(3):307–16. Available from: https://doi.org/10.1016/j.wombi.2021.04.013

[12]. Fatoye F, Gebrye T, Arije O, Fatoye CT, Onigbinde O, Mbada CE. Economic Impact of COVID-19 Lockdown on Households. Pan Afr Med J [Internet]. 2021;40(225):1–9. Available from: https://www.panafrican-med-journal.com//content/article/40/225/full

[13]. Ismail MK, Kumaran VV, Munawwarah SN, Muhamad MZ, Sarifuddin S. COVID-19 Outbreak: An Analysis of Malaysian Household Income Class during Movement Control Order (MCO). Estud Econ Apl. 2021;39(4).

[14]. Morgan PJ, Trinh LQ. Impacts of COVID-19 on Households in ASEAN Countries and Their Implications for Human Capital Development [Internet]. Asian Development

For peer review only

For peer review only - http://bmjopen.bmj.com/site/about/guidelines.xhtml

Bank Institute - ADBI Working Paper Series. 2021. Available from: https://www.adb.org/publications/impacts-covid-19-households-asean-countries

[15]. Gunarathne SP, Wickramasinghe ND, Agampodi TC, Prasanna IR, Agampodi SB. The magnitude of out-of-pocket expenditure for antenatal care in low and middle-income countries: A systematic review and meta-analysis. Int J Health Plann Manage. 2022;(August):1–25.

[16]. World Bank Group. World Bank Country and Lending Groups. Country Classification. 2019. p. 7677.

[17]. Family Health Bureau: Ministry of Health. Maternal Care Package: A Guide to Field Health Care Workers. 1st ed. Colombo: Family Health Bureau, Ministry of Health, Sri Lanka; 2011.

[18]. Agampodi T, Amarasinghe G, Wickramasinghe N, Warnasekara J, Perez-Escamilla R, Agampodi S. Maternal and child health and nutrition (MCHN) policies and programs in Sri Lanka: a paradigm for public health in Asia. Sri Lanka J Med. 2021;30(2):70–87.

[19]. Family Health Bureau: Ministry of Health - Sri Lanka. Percentage of pregnant mothers registered in 2021 [Internet]. 2022 [cited 2022 Oct 22]. Available from: https://fhb.health.gov.lk/index.php/en/

[20]. Souza J, Tunçalp Ö, Vogel J, Bohren M, Widmer M, Oladapo O, et al. Obstetric transition: the pathway towards ending preventable maternal deaths. BJOG An Int J Obstet Gynaecol. 2014;121:1–4.

[21]. Arambepola C, Wickramasinghe ND, Jayakody S, Hewage SA, Wijewickrema A, Gunawardena N, et al. Sri Lanka's early success in the containment of COVID-19 through its rapid response: Clinical & epidemiological evidence from the initial case series. PLoS One [Internet]. 2021;16(7 July):1–13. Available from: http://dx.doi.org/10.1371/journal.pone.0255394

[22]. Family Health Bureau: Ministry of Health and Indigenous Medical Service SL. Interim guidelines on maternal and child health services during CURFEW PERIOD during the outbreak of COVID-19 infection (Date:2020 March 27). Colombo, Sri Lanka; 2020.

For peer review only

For peer review only - http://bmjopen.bmj.com/site/about/guidelines.xhtml

[23]. Family Health Bureau: Ministry of Health and Indigenous Medical Service SL. Annual Report on Family Health Family Health Bureau. Colombo, Sri Lanka; 2018.

[24]. Patabendige M, Wanniarachchi D, Weerasinghe M, Ruwanpathirana P, Jayasundara D, Jayawardane A. The sustained adverse impact of COVID-19 pandemic on mental health among pregnant women in Sri Lanka: a reassessment during the second wave. BMC Res Notes [Internet]. 2022;15(1):15–9. Available from: https://doi.org/10.1186/s13104-021-05893-1

[25]. Gunarathne SP, Wickramasinghe ND, Agampodi TC, Prasanna IR, Agampodi SB. How costly is the first prenatal clinic visit? Analysis of out-of-pocket expenditure in rural Sri Lanka - a country with free maternal health care. BMC Health Serv Res. 2021;21(974):1–13.

[26]. Gunarathne, Sajaan Praveena Wickramasinghe ND, Agampodi TC, Prasanna IR, Agampodi SB. Out-of-pocket Expenditure for Antenatal Care Instills amidst Free Health Care Provision: Evidence from a Large Pregnancy Cohort in Rural Sri Lanka. Res Sq Pri-print. 2022;1–19.

[27]. Gunarathne S, Wickramasinghe N, Agampodi T, Prasanna R, Agampodi S. Economic Status, Nutrition, and Pregnancy Cost; A Vicious Cycle in Pregnancy. Curr Dev Nutr [Internet]. 2020;4(2):996. Available from: https://academic.oup.com/cdn/article/4/Supplement_2/996/5844748?login=false

[28]. Rusniya H., Nufile AAM. The Impact of COVID-19 on the Sri Lankan Economy. Int Res J Fac Arts Cult. 2021;13(4):11–9.

[29]. Agampodi TC, Wickramasinghe ND, Prasanna RIR, Irangani MKL, Banda JMS, Jayathilake PMB, et al. The Rajarata Pregnancy Cohort (RaPCo): study protocol. BMC Pregnancy Childbirth. 2020;20(1):374.

[30]. Gunarathne SP, Wickramasinghe ND, Agampodi TC, Prasanna IR, Agampodi SB. Economic burden of out-of-pocket expenditure, productivity cost during pregnancy and COVID-19 impact on the household economy in a cohort of pregnant women in Anuradhapura District, Sri Lanka; A study protocol. F1000Research. 2022;10(May):1–20.

[31]. Department of Census and Statistics of Sri Lanka. Household Income and Expenditure

For peer review only - http://bmjopen.bmj.com/site/about/guidelines.xhtml

Survey 2016 Final Report. 2018. 6 p.

[32]. Institute for Health Policy of Sri Lanka. Data and Statistics. 2020. p. 2020.

[33]. Jayasinghe I, Wickramasinghe Y, Kurera DM, Samarakoon N, Rupasinghe I, Ponmalay P, et al. Feasibility of using telephone interviews and internet-based message services during the COVID-19 pandemic in rural Sri Lanka: experiences of the Rajarata Pregnancy Cohort. Rural Remote Health. 2022;22.

[34]. Exchange Rates UK. US Dollar to Sri Lankan Rupee Spot Exchange Rates for 2019 and 2020 [Internet]. USD LKR Historical Chart for Years 2019 & 2020. 2020 [cited 2022 Jul 12]. Available from: https://www.exchangerates.org.uk/USD-LKR-spot-exchange-rates-history-2020.html

[35]. Tusl M, Kerksieck P, Brauchli R, Bauer GF. Perceived impact of the COVID-19 crisis on work and private life and its association with mental well-being and self-rated health in German and Swiss employees: a cross-sectional study. BMC Public Health. 2021;21(741):1–21.

[36]. Rauf N, Zulfiqar S, Mumtaz S, Maryam H, Shoukat R, Malik A, et al. The impact of the covid-19 pandemic on pregnant women with perinatal anxiety symptoms in Pakistan: A qualitative study. Int J Environ Res Public Health. 2021;18(16).

[37]. Shafi M, Liu J, Jian D, Rahman IU, Chen X. Impact of the COVID-19 pandemic on rural communities: A cross-sectional study in the Sichuan Province of China. BMJ Open. 2021;11(8).

[38]. Almeida V, Barrios S, Christl M, De Poli S, Tumino A, van der Wielen W. The impact of COVID-19 on households´ income in the EU. J Econ Inequal. 2021;19(3):413–31.

[39]. Chandra A, Sarda R, Kumar A, Bir M, Parija PP, Pal A, et al. Impact of COVID‑19 on the patients' income and work in Delhi, India. J Fam Med Prim Care [Internet]. 2021;10:3047–50. Available from: http://www.jfmpc.com/article.asp?issn=2249-4863;year=2017;volume=6;issue=1;spage=169;epage=170;aulast=Faizi

[40]. Kansiime MK, Tambo JA, Mugambi I, Bundi M, Kara A, Owuor C. COVID-19 implications on household income and food security in Kenya and Uganda: Findings from a rapid assessment Monica K. Kansiime a,⇑, Justice A. Tambo b, Idah Mugambi

For peer review only - http://bmjopen.bmj.com/site/about/guidelines.xhtml

a, Mary Bundi a, Augustine Kara c, Charles Owuor d a CABI Africa, P.O. Box 633-00621. 2020;(January).

[41]. Syafiq A, Fikawati S, Gemily SC. Household food security during the COVID-19 pandemic in urban and semi-urban areas in Indonesia. J Heal Popul Nutr [Internet]. 2022;41(1):1–8. Available from: https://doi.org/10.1186/s41043-022-00285-y

[42]. Murakami E. Immediate impacts of the COVID-19 pandemic on household economic activities and food security in Tajikistan [Internet]. Vol. 6, Economics of Disasters and Climate Change. Springer International Publishing; 2022. 259–291 p. Available from: https://doi.org/10.1007/s41885-021-00104-4

[43]. Kittiprapas S. Socioeconomic impacts of the COVID-19 pandemic on the vulnerable households: empirical evidence from slum areas of Bangkok city. Cogent Soc Sci [Internet]. 2022;8(1). Available from: https://doi.org/10.1080/23311886.2022.2074111

[44]. Celik B, Ozden K, Dane S. The Impact of COVID-19 on Household Economy and Consumption Preferences: An International Survey. J Glob Econ Bus [Internet]. 2020;01(02):91–115. Available from: http://www.journalsglobal.com/index.php/jgeb/article/view/29

[45]. Gunarathne S, Wickramasinghe N, Agampodi T, Prasanna I, Agampodi S. Nutritional Status and Additional Expenditure on Food During Pregnancy: Evidence From the Rajarata Pregnancy Cohort. Curr Dev Nutr [Internet]. 2021;5(2):754. Available from: https://academic.oup.com/cdn/article/5/Supplement_2/754/6293027

[46]. Gunarathne SP, Amarasinghe G, Agampodi TC, Wickramasinghe ND, Mendis V, Prasanna IR, et al. Does Being Diagnosed As Anemic in Early Pregnancy Have an Impact on the Household Expenditure for Food? Evidence From a Maternal Cohort in Rural Sri Lanka. Curr Dev Nutr [Internet]. 2022;6(1):654. Available from: https://academic.oup.com/cdn/article/6/Supplement_1/654/6606330

[47]. Gunarathne S, Wickramasinghe N, Agampodi T, Prasanna I, Agampodi S. Household Food Expenditure Share During Pregnancy: Evidence From Rural Sri Lanka. Curr Dev Nutr [Internet]. 2021;5(5):753. Available from: https://academic.oup.com/cdn/article/5/Supplement_2/753/6292904?login=false

[48]. Agampodi TC, Hromi-Fiedler A, Agampodi SB, Amarasinghe GS, Wickramasinghe

For peer review only - http://bmjopen.bmj.com/site/about/guidelines.xhtml

ND, Jayasinghe IU, et al. A self-applied valid scale for rapid tracking of household food insecurity among pregnant women in Sri Lanka. Matern Child Nutr. 2021;17(3):1–7.

[49]. Padhye R, Purushotham A, Paul M, Sardeshpande N, Ballala R, Dhar S, et al. Accessing Maternal Health Care in the Midst of the COVID-19 Pandemic: A Study in Two Districts of Assam, India. Front Glob Women's Heal. 2022;3(March):1–9.

[50]. Bailey V. Out-of-Pocket Healthcare Spending Increased Leading Up to Pandemic [Internet]. PRACTICE MANAGEMENT NEWS. 2022 [cited 2022 Oct 24]. Available from: https://revcycleintelligence.com/news/out-of-pocket-healthcare-spending-increased-leading-up-to-pandemic

[51]. Rabbani U, Saigul AA, Sulaiman A, Ibrahim TH. Impact of COVID-19 on Antenatal Care Utilization Among Pregnant Women in Qassim, Saudi Arabia. Cureus. 2021;13(11).

[52]. Banke-Thomas A, Semaan A, Amongin D, Babah O, Dioubate N, Kikula A, et al. A mixed-methods study of maternal health care utilisation in six referral hospitals in four sub-Saharan African countries before and during the COVID-19 pandemic. BMJ Glob Heal. 2022;7(2):1–14.

[53]. Sinha B, Dudeja N, Mazumder S, Kumar T, Adhikary P, Roy N, et al. Estimating the Impact of COVID-19 Pandemic Related Lockdown on Utilization of Maternal and Perinatal Health Services in an Urban Neighborhood in Delhi, India. Front Glob Women's Heal. 2022;3(March):1–10.

[54]. Muhaidat N, Fram K, Thekrallah F, Qatawneh A, Al-Btoush A. Pregnancy during COVID-19 outbreak: The impact of lockdown in a middle-income country on antenatal healthcare and wellbeing. Int J Womens Health. 2020;12:1065–73.

[55]. Agampodi TC, Rheinländer T, Agampodi SB, Glozier N, Siribaddana S. Social capital and health during pregnancy; An in-depth exploration from rural Sri Lanka. Reprod Health. 2017;14(1):1–19.

For peer review only - http://bmjopen.bmj.com/site/about/guidelines.xhtml

Rajarata University of Sri Lanka

Faculty of Medicine & Allied Sciences

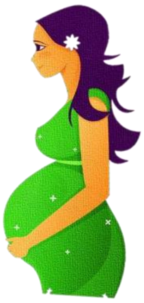

“Generating Evidence for Practice”

Rajarata Pregnancy Cohort (RaPCo)

Telephone Interview Guide

For peer review only

Maternal and Child Health Research Unit

Department of Community Medicine

Impact of COVID-19 Pandemic on Health Service Utilization and Household Economy of Pregnant and Postpartum Women: A Cross-Sectional Study from Rural Sri Lanka

Instructions for administering the questionnaire

- There are two main parts in this questionnaire. Part I (Economic Impact of COVID-19) includes five questions, and Part II (Health Service and Assistance during COVID-19 outbreak) contains nine questions.

- Most questions included here are close-ended; some were made for short answers and expected to be completed within 10-15 minutes.

- The interviewer must directly enter the answers to the questions below into the MS Excel data sheet.

- Please follow the following procedure to contact pregnant and postpartum women.

  First day - only two attempts should be made during the same day.

  Another day - Needs to be carried out for the women who could not contact during the first round, and only two attempts need to be made per day.

- After contacting, please introduce yourself and ask whether it is an appropriate time to talk over the phone. If not, politely apologize and ask for a possible time.

  "Good morning/Good afternoon!!! I am.............................(name of the interviewer) and expect to conduct a telephone interview attached to the Faculty of Medicine and Allied Sciences, Rajarata University of Sri Lanka. We are conducting this survey to find out whether the COVID-19 pandemic affected the household economy and health service utilization of pregnant and postpartum women in rural Sri Lanka. Is this an appropriate time to talk?"

- Obtain verbal consent to conduct the interview and give those about two minutes to ask for any clarifications.

- If the respondent agrees to participate in the survey, start asking questions in the questionnaire. If the respondent refuses to participate in the survey at the beginning or middle of the interview, thank her and stop the discussion.

- Please read each question as written in the questionnaire and ask the questions in the order they appear.

For peer review only - http://bmjopen.bmj.com/site/about/guidelines.xhtml

- At the end of the questionnaire, thank the respondent for her cooperation and the information provided.

- At the end of the interview, it is essential to review the questionnaire to verify that all the questions were asked and each has an answer unless the respondent has refused to answer particular questions. If any question has been missed, you should ask it the relevant respondent again.

Questions

Part I – Economic Impact of COVID-19 Outbreak

1. What is your monthly income during the COVID-19 outbreak?

2. Did your family face any income reduction during the COVID-19 outbreak?

3. Could you please explain how much income was reduced monthly during the COVID-19 outbreak?

4. Did your family receive the financial aid of Rs.5,000.00 the Sri Lankan government provided monthly?

5. Can you state around how much did you or your family spend on monthly pregnancy-related nutritional expenditures?

Part II – Health Service and assistance during COVID-19 Outbreak

1. Did you miss clinic visits during the COVID-19 outbreak?

2. If yes, please indicate the number of missed clinics.

3. Did a Public Health Midwife (PHM) visit you or check up on phone calls during the COVID-19 outbreak?

4. If yes, how many times per month?

5. How was your satisfaction level with the service provided by PHM during the COVID-19 outbreak?

    - Strongly satisfied
    - Satisfied
    - Average

For peer review only

- Not satisfied
- Strongly unsatisfied

6. Was your mode of childbirth changed due to the COVID-19 outbreak?

7. If yes, what was your planned mode, and what was the changed mode?

8. Was your place of childbirth changed due to the COVID-19 outbreak?

9. How was your satisfaction level with the support from your husband/family and neighbours during the COVID-19 outbreak?

    - Strongly satisfied
    - Satisfied
    - Average
    - Not satisfied
    - Strongly unsatisfied

For peer review only

For peer review only - http://bmjopen.bmj.com/site/about/guidelines.xhtml

Supplementary Table 1: Socio-demographic and economic characteristics of the study sample

Characteristic		Delivered during the COVID-19 pandemic (n=807, 55.3%)	Delivered before the COVID-19 pandemic (n=653, 44.7%)	Statistically significant difference/ associations*
Age of the pregnant women [Mean (SD)] in years		27.8 (5.1)	28.5 (5.9)	U=187,609.00 p=0.330
Ethnicity [n (%)]	Sinhalese	542 (85.1)	699 (86.9)	X^2 [df=1]=1.022 p=0.312
	Other minority ethnic groups[1]	95 (14.9)	105 (13.1)	
Religion [n (%)]	Buddhist	542 (85.1)	685 (85.2)	X^2 [df=1]=0.004 p=0.952
	Other religions[2]	95 (14.9)	119 (14.8)	
Educational status [n (%)]	Primary education	4 (0.7)	9 (1.2)	X^2 [df=3]=3.221 p=0.359
	Junior secondary education	21 (3.5)	39 (5.0)	
	Senior secondary education	484 (80.0)	612 (79.2)	
	Higher education	96 (15.9)	113 (14.6)	
Daily wage group [n (%)]	Yes	130 (21.0)	98 (18.7)	X^2 [df=1]=0.909 p=0.340
	No	489 (79.0)	425 (81.3)	
Income-generating members in the household	Only one person	683 (80.6)	379 (81.3)	X^2 [df=1]=0.093 p=0.760
	More than one person	164 (19.4)	87 (18.7)	
Income status in relation to the poverty line [n (%)][3]	Households below the national poverty line	105 (13.0)	68 (10.4)	X^2 [df=1]=0.632 p=0.260
	Households below the extreme poverty line	70 (8.7)	42 (6.4)	X^2 [df=1]=0.528 p=0.324
Per visit pregnancy expenditure during the first trimester (USD)	Mean (SD)	5.98 (7.77)	5.71 (7.50)	U=205,265.50 p=0.503
	Median (IQR)	4.05 (0.89-7.95)	3.59 (0.88-7.75)	
Per visit pregnancy expenditure during the second trimester	Mean (SD)	5.90 (6.99)	5.79 (6.90)	U=168,747.00 p=0.605
	Median (IQR)	3.50 (0.84-8.29)	3.48 (1.03-8.54)	

For peer review only

(USD)				

Note: [1]Tamil, Moor, Malays, and others, [2]Catholic/Christian, Hindu, Islam, and others, [3]Income status before COVID-19 pandemic, *The statistical significance difference/association of socio-demographic, economic, and health information was tested between women delivered during and before the COVID-19 pandemic, Mann-Whitney U test was used for continues variables, and Chi-square goodness of fit test was used for categorical variables

For peer review only

Supplementary Table 2: COVID-19 impact on household income and status of receiving financial aids

Description			Delivered during COVID-19 pandemic [n=648, 55.4%]	Delivered before COVID-19 pandemic [n=524, 44.7%]
Impact on household income	Families affected by income reduction [n (%)]	Yes	342 (52.8)	250 (47.7)
		No	306 (47.2)	274 (52.3)
	Reduced amount (USD)	n (%)	342 (52.8)	250 (47.7)
		Mean (SD)	124.50 (85.19)	114.65 (76.93)
		Median (IQR)	106.20 (53.10-185.84)	106.20 (53.10-159.29)
	Percentage of reduced income over household income (%)		74.5	74.8
Monthly household income (USD) changes	n (%)		342 (52.8)	250 (47.7)
	Income before COVID-19	Mean (SD)	277.96 (194.07)	224.41 (179.46)
		Median (IQR)	212.39 (159.29-318.59)	185.84 (143.37-238.94)
	Income during COVID-19	Mean (SD)	172.89 (104.91)	154.85 (78.94)
		Median (IQR)	159.29 (106.20-238.94)	159.29 (106.20-212.39)
	Statistically significant difference[1]		Z=-6.317 p<0.001	Z=-6.314 p<0.001
Households pushed into poverty [n (%)]	National poverty line		31 (4.8)	25 (4.8)
	Extreme poverty line		27 (4.2)	20 (3.8)
Financial aid provided by the government (USD 26.55)	Received financial assistance among families pushed into poverty [n (%)]		0	2 (4.8)
	Received financial assistance among affected families [n (%)]		12 (3.5)	49 (19.6)
	Percentage of received financial aid over affected income (%)		47.5	42.7
	Percentage of received financial aid		14.4	28.4

For peer review only

	over household income (%)		
	Percentage of household expenditure covered by the received financial aid (%)	31.8	24.2

Note: [1]Wilcoxon signed ranks test

For peer review only

Supplementary Table 3: Third trimester per visit pregnancy expenditure between women delivered during and before the COVID-19 pandemic

Cost [USD]	Summary Statistics	Delivered during COVID-19 Pandemic[1] [n=648, 55.4%]	Delivered before COVID-19 Pandemic[2] [n=524, 44.7%]	Statistically significant difference[3]
Cost of medicine	Mean (SD)	2.04 (3.08)	2.72 (3.76)	U=3,868.5
	Median (IQR)	1.06 (0.35-2.36)	1.42 (0.62-3.54)	p=0.050*
Cost for consultation	Mean (SD)	7.33 (3.84)	6.63 (3.08)	U=3,913.50
	Median (IQR)	6.90 (5.31-7.96)	6.70 (5.31-7.67)	p=1.164
Cost for laboratory investigation	Mean (SD)	2.17 (1.92)	2.82 (1.34)	U=48.0
	Median (IQR)	1.27 (0.93-2.65)	2.65 (2.02-3.85)	p=0.196
Cost for travelling	Mean (SD)	1.16 (2.02)	0.98 (1.32)	U=10,761.00
	Median (IQR)	0.53 (0.27-1.06)	0.53 (0.29-0.88)	p=0.941
Cost for food and refreshments	Mean (SD)	1.06 (1.29)	1.09 (1.39)	U=6,579.00
	Median (IQR)	0.66 (0.35-1.33)	0.66 (0.40-1.33)	p=0.668
Cost for accompanying person	Mean (SD)	0.85 (0.82)	1.39 (1.76)	U=925.5
	Median (IQR)	0.53 (0.40-0.93)	0.66 (0.53-1.33)	p=0.069*
Other costs	Mean (SD)	1.48 (1.86)	0.60 (0.50)	U=201.5
	Median (IQR)	0.53 (0.18-1.95)	0.60 (0.18-0.88)	p=0.181
Direct medical out-of-pocket expenditure	Mean (SD)	6.90 (6.34)	8.17 (6.72)	U=7,399.5
	Median (IQR)	6.64 (2.04-8.67)	7.06 (3.50-10.86)	p=0.095*
Direct non-medical out-of-pocket expenditure	Mean (SD)	1.94 (2.39)	2.29 (3.85)	U=14,406.0
	Median (IQR)	0.91 (0.53-2.27)	1.25 (0.44-2.43)	p=0.903
Per visit out-of-pocket expenditure in the 3rd trimester	Mean (SD)	7.26 (8.97)	7.01 (8.54)	U=19,668.00
	Median (IQR)	4.73 (1.02-9.64)	4.82 (0.73-10.19)	p=0.771

Note: [1]The cost indicated here was incurred during the COVID-19 pandemic, [2]The cost indicated here was incurred before the COVID-19 pandemic, [3]Mann-Whitney U test, *Statistically significant 0.1 level of significance

For peer review only

Supplementary Table 4: Impact of COVID-19 on health service utilization of pregnant and postpartum women and support received by PHM and family

Description			Delivered during COVID-19 pandemic [n=732, 55.7%]	Delivered before COVID-19 pandemic [n=583, 44.3%]
Clinic visits[1]	Whether missed clinics [n (%)]	Yes	104 (14.2)	40 (6.9)
		No	628 (85.8)	543 (93.1)
	Number of missed clinics [median (IQR)]		1 (1-2)	1 (1-2)
Field health services from PHM	Whether PHM visited or did phone check-ups to see the pregnant women/infant [n (%)]	Yes	670 (91.5)	500 (85.8)
		No	62 (8.5)	83(14.2)
	Number of visits per month [median (IQR)]		2 (1-3)	2 (1-3)
	Service provided by the PHM [n (%)]	Strongly satisfied	4 (0.5)	3 (0.5)
		Satisfied	667 (91.2)	429 (73.6)
		Average	50 (6.8)	138 (23.7)
		Not satisfied	8 (1.1)	10 (1.7)
		Strongly not satisfied	3 (0.4)	3 (0.5)
Childbirth	Whether changed the mode of childbirth [n (%)]	Yes	4 (0.5)*	-
		No	728 (99.5)	-
	Whether changing the place of childbirth [n (%)]	Yes	14 (1.9)**	-
		No	718 (98.1)	-
Support from husband/family and neighbours received during the pandemic	Very satisfied		4 (0.5)	12 (2.1)
	Satisfied		650 (88.8)	442 (75.7)
	Average		56 (7.7)	105 (18.0)
	Dissatisfied		15 (2.0)	12 (2.1)

For peer review only

[n (%)]	Very dissatisfied	7 (1.0)	12 (2.1)

Note: [1]Indicated clinic visits are antenatal clinic visits for the women delivered during COVID-19 and postnatal/child health clinics for women delivered before COVID-19, *Number of pregnant women expected to change the MOD, **Number of pregnant women expected to change the place of childbirth

For peer review only

STROBE Checklist for Cross-sectional Studies

Item	Item No	Page Number	Line Number
**Title and Abstract**			
Title	1	1	3-4
		3	45-46
Abstract		3-4	47-85
**Introduction**			
Background/rationale	2	5-7	112-184
Objectives	3	7	182-184
**Methods**			
Study design	4	7	186-187
Setting	5	7	189-197
Participants	6	7-8	198-215
Variables	7	9-10	242-267
Data sources	8	8	216-225
Measurement		9-10	237-267
Bias	9	8	226-232
Study size	10	11	293-299
Quantitative variables	11	9-10	242-267
Statistical methods	12	10	268-281
**Results**			
Participants	13	11	293-299
Descriptive data	14	11-12	300-307
Outcome data	15	12-15	308-370
Main results	16	12-13	308-335
		14-15	347-370
Other analyses	17	14	336-346
**Discussion**			
Key results	18	15-16	372-386
Limitations	19	4	93-99
Interpretation	20	16-17	387-427
		17-18	428-449
Generalizability	21	18	450-458
Funding	22	18	466-470

For peer review only
